# Micromagnetic Design of Skyrmionic Materials and Chiral Magnetic Configurations in Patterned Nanostructures for Neuromorphic and Qubit Applications

**DOI:** 10.3390/nano12244411

**Published:** 2022-12-10

**Authors:** Roxana-Alina One, Sever Mican, Angela-Georgiana CimpoesȖu, Marius Joldos, Romulus Tetean, Coriolan Viorel Tiușan

**Affiliations:** 1 Department of Condensed Matter Physics and Advanced Technologies, Faculty of Physics, Babeș-Bolyai University of Cluj-Napoca, 400084 Cluj-Napoca, Romania; 2 Computer Science Department, Faculty of Automation and Computer Science, Technical University of Cluj-Napoca, 400027 Cluj-Napoca, Romania; 3 National Center of Scientific Research, 54500 Vandoeuvre-lès-Nancy, France

**Keywords:** skyrmions, nanomaterials, micromagnetism, spintronics, perpendicular magnetization, Dzyaloshinskii–Moriya asymmetric exchange, patterned magnetic nanostructures

## Abstract

Our study addresses the problematics of magnetic skyrmions, nanometer-size vortex-like swirling topological defects, broadly studied today for applications in classic, neuromorphic and quantum information technologies. We tackle some challenging issues of material properties versus skyrmion stability and manipulation within a multiple-scale modeling framework, involving complementary ab-initio and micromagnetic frameworks. Ab-initio calculations provide insight into the anatomy of the magnetic anisotropy, the Dzyaloshinskii–Moriya asymmetric exchange interaction (DMI) and their response to a gating electric field. Various multi-layered heterostructures were specially designed to provide electric field tunable perpendicular magnetization and sizeable DMI, which are required for skyrmion occurrence. Landau–Lifshitz–Gilbert micromagnetic calculations in nanometric disks allowed the extraction of material parameter phase diagrams in which magnetic textures were classified according to their topological charge. We identified suitable ranges of magnetic anisotropy, DMI and saturation magnetization for stabilizing skyrmionic ground states or writing/manipulating them using either a spin-transfer torque of a perpendicular current or the electric field. From analyzing the different contributions to the total magnetic free energy, we point out some critical properties influencing the skyrmions’ stability. Finally, we discuss some experimental issues related to the choice of materials or the design of novel magnetic materials compatible with skyrmionic applications.

## 1. Introduction

One of the most recent magnetic paradigms in information and communication technologies (ICT) is based on the use of magnetic skyrmions. These vortex-like swirling topological defects in the magnetization texture are particularly interesting due to their extremely small size, and low level of energy consumption when manipulated to change their state that can encode either classic, neuromorphic or quantum information. The request for low energy consumption represents a major challenge of next-generation ICT devices, having in view the exponential increase in energy consumption in this area. In 2018, ICT accounted for between 5% and 9% of total electricity consumption worldwide [1]. Widely cited forecast sources suggest an acceleration of the total energy demand in the ICT area in relation to new blockchain applications (e.g., cryptocurrencies), and to emerging neuromorphic and quantum technologies. Large data centers and wireless and wired networks demand more and more energy compared to consumer devices such as televisions, computers, mobile phones. An optimistic prediction expects that in 2030, more than 20% of the global electricity consumption, about 9000 Terawatt (TWh) hour, will come from the ICT sector [1]. This trend is clearly distinguishable when looking at the data provided by Google on the *web*, regarding the energy consumption for the search query: an average Google search event requests about 0.0003 kWh of energy, the equivalent of turning a 60 W light bulb for about 17 s, 30 searches cost the energy of boiling one litter of water, the total Google search consumption in 2015 was about 5.7 TWh, similar to the total annual energy consumption of San Francisco (5.8 TWh). This trend tripled between 2015 and 2020, the analysis illustrating a clear exponential increase. The increase will be further amplified by the increase in the total number of bits produced and manipulated annually on Earth [2], today about 10^21^. In the next 300 years, the power requested to sustain the digital production is expected to exceed 18.5 × 10^15^ W, i.e., the total planetary power consumption, today. Therefore, aside from the existing global challenges related to the environment, climate, population, health, energy, food and security, it is obvious that the energetic efficiency of any device used in ICT represents a major request. New concepts in data storage and the manipulation area must emerge. Spintronics should subscribe to this paradigm and the magnetic skyrmions are particularly suitable for that. They have the advantage of being particularly small, can be manipulated with extremely low levels of energy when storing classic, neuromorphic or quantum information, and ultimately, bring additional functionality driven from magnetic topology issues into consumer-friendly, low-energy nanoscale electronics. Within this context, there is a strong motivation for the theoretical modeling of skyrmionic materials and devices. Our objective is to identify the critical magnetic parameters of materials for hosting and manipulating skyrmions.

In this paper, using a multiscale modeling approach, we address the problematics of skyrmion stabilization versus critical magnetic properties of nanomaterials. Using ab-initio calculations on supercells describing realistic/experimental multilayer architectures, we extract the mechanisms and magnitude of some main magnetic properties: magnetic moments and anisotropy (PMA), the Rashba contribution to the interface asymmetric exchange constant DMI, and their response to an external gating electric field. Then, micromagnetic simulations based on Landau–Lifshitz–Gilbert dynamics, led to various magnetic binary phase diagrams of magnetic nanostructures in which the final relaxed state is classified via the topological charge. The micromagnetic simulation results illustrate critical conditions in which skyrmionic states can be written by pulse currents in nano-disks via STT effects. This allows us to identify the DMI-PMA range in which skyrmionic ground states can be stabilized in nano-disks patterned from magnetic multilayers with various sizes and architectures. Furthermore, the results point out some critical parameters which determine the skyrmionic properties (size, stability in temperature, etc.) and illustrate how the skyrmions can be manipulated by external electric field gating or spin-polarized currents. Finally, we discuss some experimental issues related to the choice of materials or the design of novel magnetic materials compatible with skyrmionic applications in classic, neuromorphic and quantum information technologies.

## 2. Materials and Methods

### 2.1. General Issues about Skyrmions

Magnetic skyrmions were expected to be short-time excitations quickly collapsing on linear singularities. However, the seminal work of Bogdanov and Yablonski (1989) demonstrates that they can exist as a metastable configuration with a sizeable lifetime in some low-symmetry condensed matter systems with broken mirroring symmetry [3]. The mechanism responsible for their stabilization is the asymmetric exchange interaction for either bidimensional (2D) or tridimensional (3D) spin localized states. This is the so-called Dzyaloshinskii–Moriya interaction [4] which typically occurs in non-centrosymmetric magnetic materials (bulk DMI), at the interface between a heavy metal and a ferromagnetic thin film (interface DMI) and in some electric field gated ferromagnetic film surfaces (Rashba DMI). Even if at the beginning, the DMI has been only regarded as a higher order effect occurring between ions already coupled by super-exchange (about 10^−2^ times smaller than the direct exchange [5], the DMI was further found to be particularly important in ferromagnetic materials with a large spin-orbit coupling (SOC) $H_{SO}=\zeta \left(r\right)\vec{L}\cdot \vec{S}$ which also breaks the inversion symmetry. The corresponding interaction term in the Hamiltonian would be $H_{DMI}=\left({\vec{S}}_{1}\times {\vec{S}}_{2}\right)\cdot {\vec{D}}_{12}$, where ${\vec{D}}_{12}$ is a vector perpendicular to the plane of the spins ${\vec{S}}_{1\left(2\right)}$. This term tilts the magnetic moments away from the collinearity and its magnitude proportional to the SO interaction included in the SO coupling parameter $\zeta \left(r\right)$. The low dimensionality of magnetic nanostructures brings additional sizeable contributions to the DMI via the associated additional potential gradients and corresponding intrinsic electric fields. At the interface between magnetic and nonmagnetic materials (heavy metals, insulators, etc.) the lack of inversion symmetry leads to a gradient of the lattice potential. Moreover, in multilayered stacks, additive interface effects would provide enhanced DMI and other interesting features. For example, it is possible to tune the sign of the DMI. Because the vector ${\vec{D}}_{ij}$ is parallel to the film plane in magnetic multilayers with perpendicular magnetization, its orientation is defined by the chemical nature and electronic structure effects at the interface between the nonmagnetic and the ultrathin ferromagnet. This leads to an enhancement of the effective magnitude $\left|{\vec{D}}_{ij}\right|$ with decreasing ferromagnetic layer thickness.

Therefore, the magnetic multilayer stacks provide a large degree of freedom in skyrmion stabilization, mainly based on the sizeable interface DMI and the perpendicular magnetic anisotropy (PMA) in ultrathin magnetic films. Within this area, during the past few years, many experimental studies have been performed. They illustrated the successful stabilization of skyrmions from below to above room temperature (RT). The skyrmion stability and its size (core diameter) are controlled by the interplay between the magnitude of the DMI and the dipolar interactions. Most of the studied skyrmionic systems were based on Co, Fe and CoFeB ultrathin ferromagnetic layers, interfaced with Pt, Ta, Ir or W heavy metals and/or MgO insulator [6,7,8,9,10,11,12,13,14,15,16] (see Table 1). Additive effects, by multiple repetitions of the skyrmionic active magnetic stacks (e.g., [Ir/Co(0.6 nm)/Pt]_n = 10_) were found to enhance the thermal stability of skyrmions, even above the room temperature [9]. This represents an extremely important issue for the technological implementation in RT working ICT devices. The skyrmions can be efficiently displaced either by spin-orbit torque [6] or by spin-transfer torque (STT) effects in skyrmion racetrack memories [16] and can be created and annihilated by electric fields [17]. 

The expected application area of skyrmions is quite broad, from classic spintronic devices such as racetrack memories [16], to neuromorphic spintronics (artificial skyrmionics neurons/synapses) [18] and, as more recently claimed, to quantum spintronics applications based on skyrmionic helicity qubits [19]. A skyrmion racetrack memory is very similar to a domain wall memory [20]. Here, skyrmions are moved by STT of spin-polarized currents instead of domain walls. The presence or absence of a skyrmion, read by a fixed magnetic tunnel junction head placed near the track represents the classic 1 and 0 states. The smaller size of skyrmions compared to domain walls enables larger areal storage densities. In a neuromorphic device, e.g., skyrmionics memristor, the particle-like behavior of skyrmions and their thermal Brownian motion have strong analogies with the neurotransmitter diffusion. Skyrmions accumulating in a “reservoir” could be the spintronic analogues of leaky integrate-and-fire neurons. Moreover, nonlinear resistance changes in magnetic skyrmion systems, originating from the interplay of magnetoresistance and spin or spin–orbit torques on the skyrmions that either move or distort them, can be exploited for unconventional computing [18]. Finally, the skyrmionic quantum bit [19] takes advantage of the quantum information that a skyrmion can store in its helicity: a quantum state being an arbitrary superposition of skyrmion configurations with distinct helicities, uniformly distributed over the Bloch sphere. Moreover, antiferromagnetically coupled skyrmions in synthetic antiferromagnetic multilayers have been recently proposed as platforms for a qubit coupling scheme where the logical states can be adjusted by electric fields [19].

Independent of the type of device, the skyrmions must be manipulable for changing the information they store by external stimuli: fields, spin currents, etc. For instance, the skyrmion size can be tuned by magnetic fields [9], and their chirality and size by electric fields [21]. However, other magnetic parameters such as magnetic anisotropy (PMA), direct and asymmetric exchange (DMI), and saturation magnetization (influencing the magnetostatic energies) will also influence the skyrmion stabilization, their size and stability in temperature.

**Figure 1:**
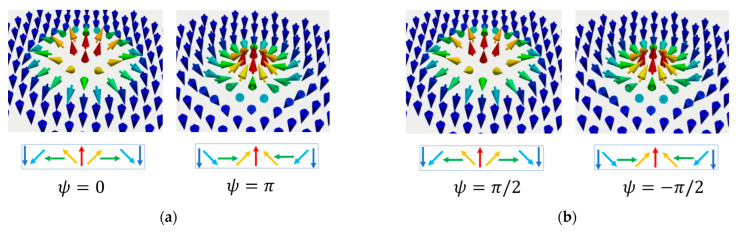
(**a**) Right- and left-handed Néel skyrmions; (**b**) Right- and left-handed Bloch skyrmions.

As magnetic textures, skyrmions resemble vortices but with the *z* component of the magnetization unit vector $m_{z}$ varying from +1 to −1. In a magnetic nanostructure, the ansatz magnetization profile of a vortex in spherical coordinates, $m=\left(\sin\theta \cos\phi ,\;\sin\theta \sin\phi ,\;\cos\theta \right);r=\left(r,\varphi \right)$ is $\theta =\theta \left(r\right),\;\phi \left(r\right)=\varphi \pm \frac{\pi }{2}$, and minimizes the volume and edge surface charges: −∇·m=0 and $m\cdot \hat{r}=0$. The sign ± in $\phi \left(r\right)$ determines the chirality of the vortex. In a skyrmion the magnetic configuration results from the competition between the exchange, anisotropy and the chiral DMI interaction and can be roughly described by an inexact but useful ansatz profile [22] $\theta_{DW}\left(r\right)=2\arctan\left[\frac{\sinh\left(R/w\right)}{\sinh r/w}\right]$ as a single 360° domain wall, with some characteristic parameters *R*, *w* being the skyrmion size and skyrmion domain wall width, respectively. To further understand the differences between skyrmions in various materials it is important to emphasize the different types of skyrmions and the microscopic origin of their stability. First, the skyrmions can be distinguished by their respective domain walls (DWs), as right- and left-handed Néel and Bloch spin structures, as illustrated by our micromagnetic simulations (Figure 1).

The main parameters of the skyrmions are:

- The topological charge or the winding number *Q =* |1| that describes how many times the magnetization vector **m** can be mapped onto a sphere: $Q=\frac{1}{4\pi }\int d^{2}x\;\vec{m}\cdot \left(\frac{d\vec{m}}{dx}\times \frac{d\vec{m}}{dy}\right)$. In saturated magnetic states *Q = 0*, *Q* = |0.5|for magnetic vortices, and more complex structures that wrapping the Bloch sphere multiple times *Q* >|1|.
- The vorticity (chirality) ω = +/−1; ω = ((*end*) − θ(*start*))/2π. The sign of ω and Q are directly related to the orientation of the skyrmion core: *P* = ω·*Q* = 1/−1 for up/down, respectively; topological charge calculated from vorticity $Q=\frac{m}{2}\left[\underset{r\to \infty }{\lim}\cos\theta \left(r\right)-\cos\theta \left(0\right)\right]$.
- The helicity is uniquely determined by the type of DMI that occurs in the energetic competition (bulk or surface): $\psi =\left[0,\pi \right]$ for a Néel skyrmion and $\psi =\left[\frac{\pi }{2},3\pi /2\right]$ for a Bloch skyrmion.

**Figure 2:**
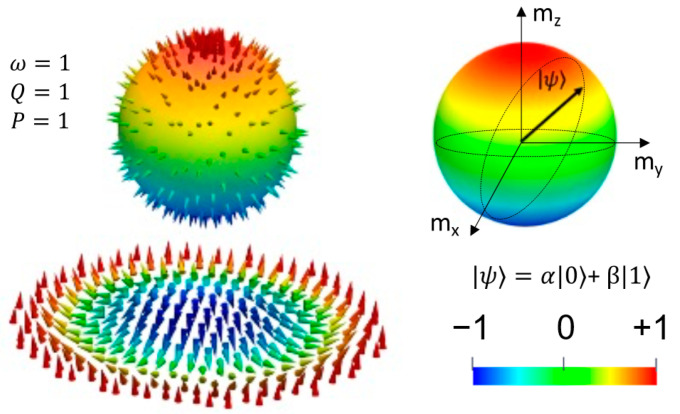
Left: Hedgehog representation of a Néel skyrmion magnetization vector field projected on the Bloch sphere. We indicated the vorticity ω, the topological charge Q and the core polarization P. Right: Bloch sphere and the helicity qubit: encoding the quantum superposition of $|0\rangle :\;m_{z}=+1$ and $|0\rangle :m_{z}=-1$: $|\psi \rangle =\alpha |0\rangle$+ $\beta |1\rangle$. The representation is issued following our micromagnetic simulations, see next paragraph.

The skyrmion parameters are linked to the generalized definition of a nanoscale skyrmion magnetization field: $m\left(r\phi \right)=\left(\begin{array}{c} \sin\Theta \left(r\right)\cos\Phi \left(\varphi \right)\\ \sin\Theta \left(r\right)\sin\Phi \left(\varphi \right)\\ \cos\Theta \left(r\right) \end{array}\right)$; $\Phi \left(r\right)=\varphi +\psi$; with ψ being the helicity. This allows the illustration of the skyrmion’s topological analogy with a sphere, the projection on the Bloch sphere of the magnetization vector field being particularly useful for understanding the helicity qubit (Figure 2).

There is a direct correlation between the winding number and the stability of the spin structures. Theoretically, the Skyrmionic winding number is an integer (positive or negative). The physical manifestation of this topological quantization is that the magnetic skyrmions are topologically protected from decaying to a uniform magnetization that has a winding number equal to zero. As we will show in the sections dedicated to results and discussion, the spins from the core of the skyrmion are protected by an energy barrier driven by the DMI from the reversed spins from the edge.

### 2.2. Ab-Initio Calculations of Magnetic Properties

The ab-initio calculations for extracting and analyzing the magnetic properties have been performed using the Full Potential Linear Augmented Plane Wave FP-LAPW Wien2k code [23]. The ab-initio results have been used, in a first step, as starting points for further micromagnetic calculations. For modeling multilayer stacks compatible with PMA and DMI sizeable effects we used supercells with typical *X*/Fe(t_Fe_)/MgO(001) configuration, where *X* = nonmagnetic metal (Au, V, Ag) or insulator (MgO). The thickness of the ferromagnetic Fe layer (*t_Fe_*) was chosen in a few monolayers (ML) range (e.g., 5ML), as required for promoting the perpendicular magnetization ground states driven by the interplay of both top and bottom interfacial PMA. The calculation of the magnetic anisotropy energy (MAE) was executed by means of a fully relativistic spin–orbit scheme using the total energy and force theorem approaches [24], both providing similar results. Using the total energy approach, including the spin-orbit, the perpendicular magnetic anisotropy (PMA) has been estimated as the total energy difference between the easy (magnetization perpendicular to the film plane) and the hard directions (in-plane magnetization). Considering the extreme sensitivity of the magnetic anisotropy energy to the *k*-space meshing, first, a convergence study of the total energy with respect to the total number of *k*-points has been thoroughly performed. 

The spin-orbit term of the non-relativistic Dirac (Pauli) Hamiltonian:

$H_{SO}=\frac{\hbar }{{\left(2m_{0}c\right)}^{2}}\vec{\nabla }V\cdot \left(\hat{\sigma }\times \vec{p}\right)$, with $\hat{\sigma }=\left(\sigma_{x},\;\sigma_{y},\;\sigma_{z}\right)$ the Pauli matrices vector and $\vec{p}$ the electron momentum moving in a potential gradient $\vec{\nabla }V$, can be expressed for 2-dimensional electronic systems with the confinement direction (e.g., *Oz* in a Cartesian system) transversal to the propagation direction (within the *xOy* plane) leading to the Rashba Hamiltonian: $H_{R}=\alpha_{R\;}\hat{\sigma }\left(\vec{k}\times {\vec{e}}_{z}\right)$ where $\alpha_{R}=\frac{\hbar^{2}}{{\left(2m_{0}c\right)}^{2}}\frac{\partial V}{\partial z}$ is the Rashba constant which is a measure of the spin-orbit interaction and ${\vec{e}}_{z}\;$the unit vector of the *Oz* (which corresponds to the electron confinement direction). According to its definition, one can conclude that $\alpha_{R}$ is proportional to the electric field $E=-\frac{\partial V}{\partial z}$ pointing along the *Oz* direction. In a multilayer stack, between two adjacent materials, such intrinsic electric fields naturally exist and lead to Rashba spin-orbit interaction effects. This bidimensional case is a good description of ultrathin films, their thickness in the few monolayer range being smaller than the quantum coherence length of electrons. In the situation when a metal-insulator (or metal semiconductor) are associated in a multilayer heterostructure, forming an interface, a depletion zone is defined with a significant interfacial electric field associated. By diagonalizing the Rashba Hamiltonian, the eigenvalues will be: $E_{\pm }\left(k_{II}\right)=\frac{\hbar^{2}k_{II}^{2}}{2m_{0}}\pm \alpha_{R}\left|k_{II}\right|$, which correspond to parabolic bands with an offset of the parabola minimum in positive (or negative) *k* values (Rashba splitting). The minimum of the parabola can be found when derivative condition is $\frac{\partial E}{\partial k}=0\Rightarrow k_{0}=\frac{m_{0}\alpha_{R}}{\hbar^{2}}$ leading to $E_{\min}=E_{0}=\frac{\hbar^{2}k_{0}{}^{2}}{2m_{0}}$ and therefore, $E_{0}=\frac{k_{0}\alpha_{R}}{2}$. Considering this expression of *E*_0_, we concluded that $\alpha_{R}$ could be calculated using the values of *E* and *k* corresponding to the minimum of the parabolic dispersion band: $\alpha_{R}=\frac{2E_{0}}{k_{0}}$. We used this expression to deduce the Rashba parameter from the Rashba parabolic offset of the bands calculated for different supercell architectures describing magnetic heterostructures. Knowing the Rashba parameter, one can calculate the Rashba contribution to the DMI as [25]: $DMI=2k_{R}A$, with *A* = the exchange stiffness of the ferromagnetic material (e.g., 21 pJ/m for Fe), $k_{R}=\frac{2\alpha_{R}m_{e}}{\hbar^{2}}$, with *m*_e_ the electron effective mass of the Rashba shifted parabolic bands.

**Figure 3:**
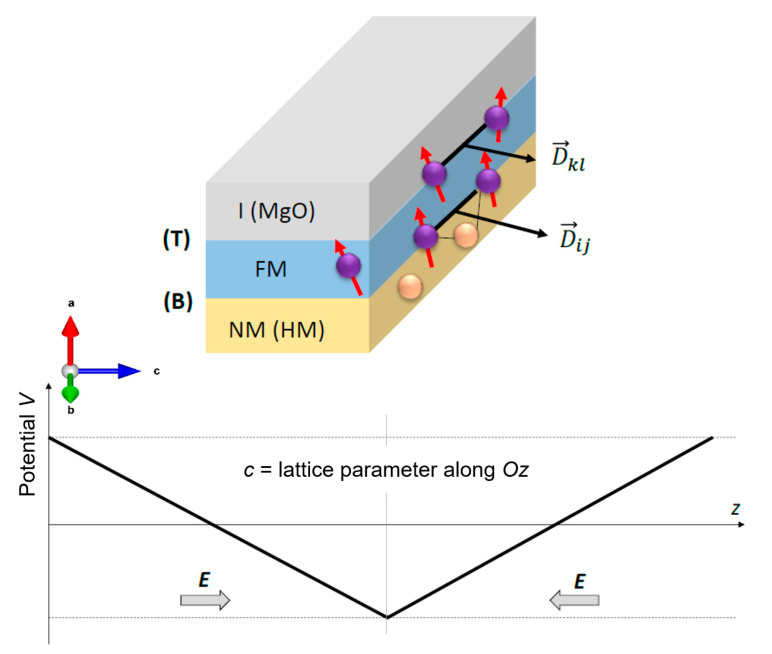
Top: Multilayer stack in which the PMA and DMA result from additive effects of the top and bottom interfaces. Bottom: zig-zag potential model used for simulating the effect of an electric field on the electronic structure of a multilayer stack (adapted from [27]).

Within these formalisms, the electric field (E-field) can be applied using a zig-zag additional potential in the Hamiltonian (Figure 3), as implemented by Stahn et al. [26]. 

Therefore, for different values of the electric field, the (perpendicular) magnetic anisotropy (PMA), and the Dzyaloshinskii–Moriya interaction (DMI) can be calculated to provide theoretical insight on their origin and electric field dependence.

### 2.3. Micromagnetic Modeling Tools

The micromagnetic simulations have been performed using the Mumax^3^ GPU accelerated code (Ghent University, Ghent, Belgium) [28], based on solving the Landau-Lifshitz-Gilbert equation:

(1) $$\frac{dm}{dt}=-\frac{\gamma }{1+\alpha^{2}}\left(m\times B_{eff}\right)-\frac{\alpha \gamma }{1+\alpha^{2}}\left[m\times \left(m\times B_{eff}\right)\right]$$

where $B_{eff}$ is the effective field, *α* is the Gilbert damping parameter, *γ* is the gyromagnetic ratio (1.75 × 10^7^ s Oe^−1^) and *μ*_0_ the vacuum permeability (4π × 10^−7^ F/m). 

The effective field can be calculated as the functional derivative of the magnetic free energy of the system *E*[**m**,t] that includes various contributions: Zeemann, demagnetizing, anisotropy, exchange: symmetric (*J*_0_) and asymmetric (DMI), STT effects, etc.: $B_{eff}=-\frac{1}{M_{s}}\frac{ðE}{ðm}$. In our calculations, we have employed an NVIDIA Geforce RTX 3070 graphic card with 5888 CUDA cores for parallel computing. The modeled systems are nanometric-size disks constituted from tri-layer stacks, like those modeled by ab-initio calculations, in which a ferromagnetic film is sandwiched between a top (T) and a bottom (B) nonmagnetic layer. Therefore, the PMA and DMI are controlled by additive interface effects (top panel of Figure 3). In the Mumax^3^ code, the perpendicular magnetic anisotropy was introduced as a 1st order uniaxial anisotropy *K*_u_ along the *Oz* (001) axis, and the DMI has been chosen to have interfacial origin (*D_ind_*), as expected for ultrathin films stacks. In the simulations, we included as variables: other specific material/multilayer stack parameters such as the *α*- Gilbert damping of FM, exchange stiffness *A_ex_*, the saturation magnetization of the FM, the shape/size of the patterned nanostructure (e.g., disk, track, …), the density of a spin-polarized current injected in a nano-disk for STT writing of skyrmions and the time length of current or gating electric field pulses. For dynamical simulations, the LLG equation has been integrated using the adaptive step RK45, the Dormand–Prince solver. For the calculations of the phase diagrams, the energy minimum of the states has been obtained using the *mumax*3 *relax* () function that disables the precession term of the LLG equation allowing the system to relax towards the minimum of energy. For including the temperature effects, a finite temperature has been set to the ground state after the relaxation. Then, the evolution/stability of the system has been studied within a finite time integration window. The classification of the final micromagnetic states has been performed by topological charge calculations: $Q=\frac{1}{4\pi }\int d^{2}x\;\vec{m}\cdot \left(\frac{d\vec{m}}{dx}\times \frac{d\vec{m}}{dy}\right)$ tested against either artificial intelligence (AI) algorithms (image recognition).

## 3. Results

### 3.1. Ab-initio Calculations of Multilayer Stacks

These calculations provide direct insight into the magnetic properties responsible for the skyrmion stabilization and their control by external stimuli, e.g., electric or magnetic fields. These parameters are the perpendicular magnetic anisotropy PMA, the asymmetric exchange DMI and the saturation magnetization, calculated as the total magnetic moment divided by the volume of the unit supercell. 

**Figure 4:**
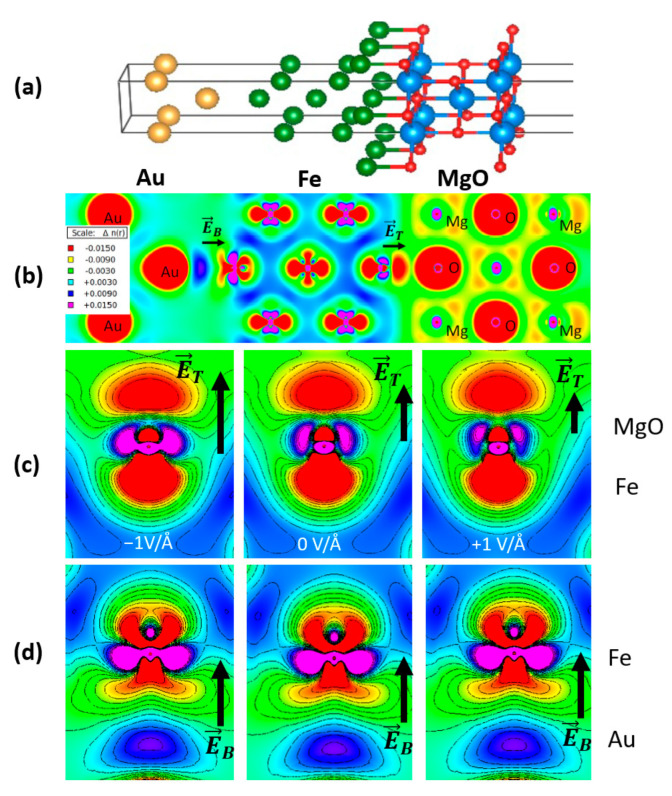
(**a**) Supercell model used in the ab-initio calculation for *X*/Fe(t_Fe_)/MgO stack. (**b**) Valence charge distribution calculated for Au/Fe(5ML)/MgO at zero electric field, corresponding to (110) section of the supercell-complete stack. (**c**) zoom at the top Fe/MgO and (**d**) bottom Au/MgO interfaces illustrating the effect of an external electric field on the top and bottom interface intrinsic fields **E**_T_ and **E**_B_ (adapted from [28]).

First, we focused on the charge depletion effects at the interfaces between the ferromagnetic film and the bottom *X* and top MgO layers. Figure 4 illustrates a typical example of charge density representation, calculated for an Au/Fe/MgO supercell. A similar analysis was performed for other bottom underlayers: e.g., *X* = V, Ag, MgO. Corresponding to this charge depletion, an intrinsic electric field would appear at each interface. Its orientation depends on the relative magnitude of the work functions in Fe and *X* and Fe and MgO, respectively. In the case of the Au/Fe/MgO stack, from the valence charge density distribution one can distinguish the intrinsic charge depletion zones at the bottom Au/Fe and top Fe/MgO interfaces, and, from the color map corresponding to the charge density variation Δ*n*, the orientation and the magnitude of the corresponding intrinsic electric fields **E**_B_ and **E**_T_, that would be responsible for the PMA and DMI. Moreover, one can qualitatively observe that a positive (negative) voltage bias has an effect, the decrease (enhancement) of the intrinsic field at the top Fe/MgO interface (Figure 4c) and, as expected, no effect for the metallic Au/Fe bottom interface (Figure 4d). It was demonstrated that this intrinsic interface electric field would be responsible for both PMA and DMI [25,29]. Moreover, as theoretically predicted by Barnes et Maekawa [30], the additive effect of the intrinsic electric fields of both top and bottom interfaces would lead to a net Rashba contribution to the PMA and DMI whose magnitude and signs can be modulated by the chemical nature of the underlayer and overlayer. Therefore, within this interface Rashba framework, one can easily understand the effect of an external gating electric field which, depending on its orientation will either enhance or decrease the value of the intrinsic interface fields and implicitly, the magnitude of the PMA and the DMI.

**Figure 5:**
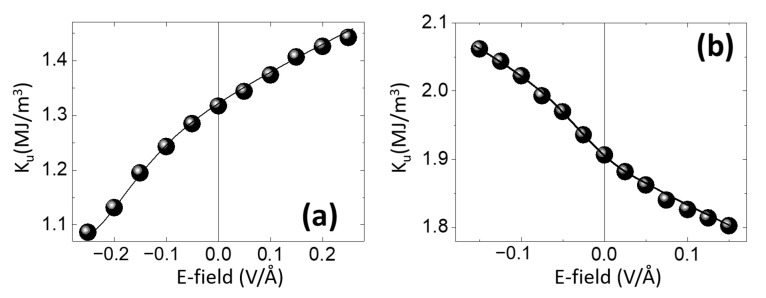
Opposite sign variation of the perpendicular magnetic anisotropy energy (*K*_u_) with an applied electric field (E-field) calculated for (**a**) V/Fe(5ML)/MgO and (**b**) Au/Fe(5ML)/MgO supercells- (adapted from [28]).

Indeed, our ab-initio calculations fully subscribe to this framework and confirm the quantum analytical predictions of Barnes et al. [30], illustrating the opposite sign variation of the PMA with the electric field, corresponding to V/Fe(5ML)/MgO and Au/Fe(5ML)/MgO systems (Figure 5). The PMA, described by the effective anisotropy constant *K*_u_ (J/m^3^), is calculated from the surface anisotropy energy *K*_s_ (J/m^2^), using the equation *K*_u_ (J/m^3^) = *K*_s_ (J/m^2^)/ t_Fe_, with t_Fe_ being the thickness of Fe (0.715 nm for 5ML). The value of *K*_s_ is obtained by dividing the total energy difference between the easy (magnetization perpendicular to the film plane) and the hard directions (in-plane magnetization) to the area of the supercell base (Figure 4a).

**Figure 6:**
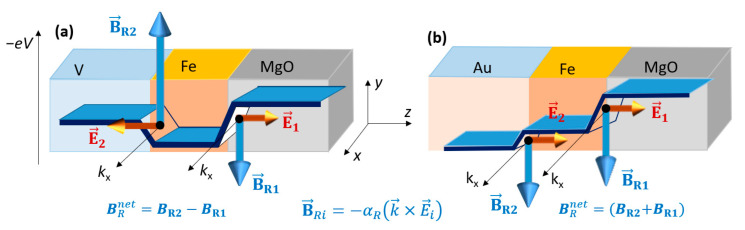
Potential profiles and Rashba magnetic fields ${\vec{B}}_{Ri}=-\alpha_{R}\left(\vec{k}\times {\vec{E}}_{i}\right)$ related to the intrinsic electric fields ${\vec{E}}_{i}$, *i* = 1,2 the top (1) = Fe/MgO and the bottom (2) = X/MgO interfaces in (**a**) V/Fe(5ML)/MgO and (**b**) Au/Fe(5ML)/MgO systems–(adapted from [28]).

This opposite behavior can be explained by considering the effect on an external electric field on the additive Rashba field of each interface in the *X*/Fe/MgO stack. In Figure 6 we illustrate the potential profiles seen by an electron in the *X*/Fe/MgO stack, considering the corresponding work functions of the three elements: *X* (V and Au), Fe and MgO. These profiles agree with the orientation of the intrinsic electric fields at each interface ${\vec{E}}_{i}$, *i* = 1,2 obtained by analyzing the interfacial depletion of the electronic charge density, similar to the situation shown in Figure 4.

**Figure 7:**
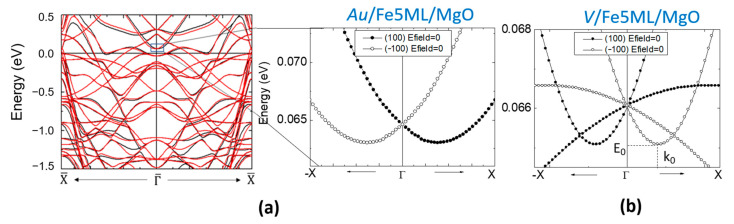
Energy bands oppositely shifted in *k*, and zooms in a narrow k and energy range (black box) - corresponding to the magnetization **M** ‖(100) and **M** ‖ ( (−100), respectively, calculated for (**a**) Au/Fe/MgO and (**b**) V/Fe/MgO systems demonstrate the opposite sign of the net Rashba field ${\vec{B}}_{R}^{net}$ in the two situations (adapted from [28]).

The corresponding interfacial Rashba magnetic fields are ${\vec{B}}_{Ri}=-\alpha_{R}\left(\vec{k}\times {\vec{E}}_{i}\right)$, the net (additive) Rashba field felt by the Fe layer, ${\vec{B}}_{R}^{net}={\vec{B}}_{1}+{\vec{B}}_{2}$ would have a magnitude whose sign would depend on the type of the *X* underlayer (as confirmed by band structure analysis depicted in Figure 7). Therefore, the effect of an external electric field would be different. Depending on the nature of *X*, a net increase/decrease in the ${\vec{B}}_{R}^{net}$ (and, consequently, of the Rashba contribution to the PMA and DMI) would be observed. In the case of Au/Fe/MgO, from the valence charge density analysis (Figure 4c) we saw that a positive bias voltage (corresponding to a negative external electric field) leads to a reduction in the top interface electric field and, consequently, to a reduction in MAE, as seen in Figure 5b).

Furthermore, the DMI can be calculated from the Rashba parameter $\alpha_{R}$, estimated from the shift in *k* of the band structure corresponding to a spin-orbit ab-initio calculation with the magnetization **M** fixed along (100) and (−100) directions (Figure 7).

**Figure 8:**
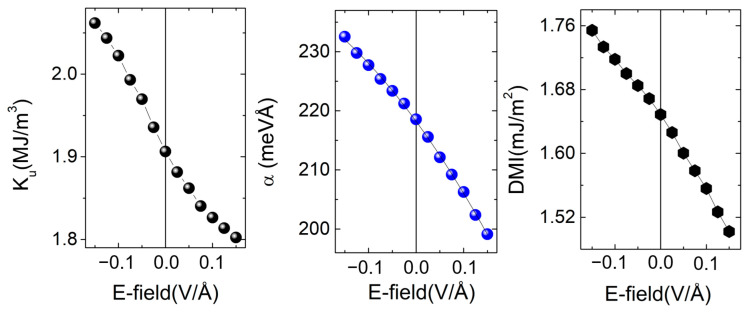
Electric field dependence of the surface anisotropy *K*_u_ (PMA), $\alpha_{R}$ and DMI, here for the Au/Fe(5ML)/MgO system–(adapted from [28]). The zero field values: *K*_u_(E-field = 0) = 1.91 MJ/m^3^ and DMI (E-field = 0) = 1.67 mJ/m^2^ will be further used in micromagnetic simulations (see Section 3.2.2).

Considering this framework, our scope was to compute, explain and correlate the sign, the magnitude and the electric field dependence of the anisotropy, the Rashba coefficient $\alpha_{R}$, and the Dzyaloshinskii–Moriya (DMI) interaction parameter in different types of *X*/Fe/MgO(001) systems in which the variable layer was *X* (where X = V, Au, Ag, Pd, MgO, etc.). A typical example of such kind of result is depicted in Figure 8, corresponding to the ab-initio analysis of the Au/Fe(5ML)/MgO system.

We also studied the influence of a monolayer of different metals, e.g., Pt, Au or Pd inserted at the top interface between Fe and MgO. We observed that in the case of a Pt, the PMA, the DMI and their variation with respect to an external electric field are always enhanced, independently of the nature of *X*. The most significant effect has been found for the Au/Fe(5ML)/Pt(1ML)/MgO system. Here, the Pt ad-monolayer amplifies the PMA by an order of magnitude, from 1.91 to 15.77 MJ/m^3^, the DMI from 1.67 to 2.27 mJ/m^2^, and the variation of PMA with E-field roughly by a factor of 2. These results indicate promising strategies for tailoring the PMA, DMI and their response to an electric field, very important issues for micromagnetic properties and control, as we will illustrate later. 

Therefore, the ab-initio calculations will be very important for a further micromagnetic analysis of skyrmionic nanomaterials. The opposite sign of the Rashba coefficient $\alpha_{R}$ in different systems would determine the opposite signs of the Dzyaloshinskii–Moriya interaction (DMI). This is particularly important because the sign of the DMI determines the chirality of the chiral structures (domain-walls, skyrmions) and, in correlation with the orientation of the core polarization, their velocity direction driven by spin currents (STT). Moreover, the sign and the magnitude of the PMA and DMI variation with an electric field determines the possible trajectory for the displacement within the magnetic parameters phase diagrams (discussed in the next section) under the applied electric field, in view of skyrmions stabilization and manipulation for various applications.

### 3.2. Micromagnetic Design of Skyrmionic Materials

This section addresses the question about the critical material magnetic parameters for injecting, hosting as ground states, and manipulation of skyrmions in patterned nanostructures. Using micromagnetic simulations, we calculated different types of phase diagrams of final magnetic states in nanometric-size patterned disks. First, we used as input the magnetic parameters obtained from ab-initio calculations for the Au/Fe/MgO system, in which the PMA, DMI and their variation with gating electric fields are controlled by interfacial cumulative effects (Figure 6b). Within these circumstances, we calculated the phase diagrams for skyrmion injection by spin-transfer-torque (STT) effects related to spin-polarized currents in patterned nanodisks of various diameters. Second, the skyrmionic injection by STT has been addressed via extended phase diagrams, in which the current pulse parameters have been fixed and the material parameters, PMA and DMI, have been varied in a wider range, to cover other potential skyrmionic materials. Then, to get a deeper understanding of mechanisms and magnetic free-energy contributions responsible for the stabilization and the stability of skyrmions, our study has been further extended to phase diagrams of ground states, in nanodisks with different sizes and shapes. We succeeded to identify the occurrence of various micromagnetic chiral states and analyzed the skyrmions stability/evolution as a function of magnetic and geometric parameters: PMA, DMI, M_s_ and disk size. We briefly extrapolated the phase diagrams to the case of antiferromagnetically coupled skyrmions that could be stabilized in synthetic antiferromagnetic materials with interesting perspectives for racetrack memories and coupled qubits applications. Furthermore, we discussed some skyrmion manipulation issues: in nano-oscillators and racetracks, we illustrate the possibility to generate multi-skyrmionic ground states interesting for memristor functions in neuromorphic devices. Finally, we address some issues related to the skyrmionic state manipulation (core polarization and chirality by external gating electric fields). The choice of the diameter size of the disk in most simulations (90 nm) has been optimized as a compromise between the following issues: (i) our initial fixed target, to have nanometric size skyrmions in materials with experimentally achievable DMI (having in view our observation concerning the inverse proportionality between the size of a skyrmion and the DMI required to stabilize it), (ii) the efficiency and versatility of the skyrmionic injection by STT in larger disks increases with the disk size.

#### 3.2.1. Injection of Skyrmions by STT in Patterned Nanodisks

**Figure 9:**
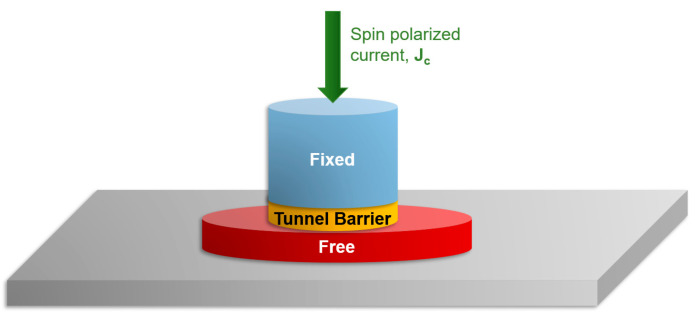
Simulation geometry for skyrmion injection in nano-disks as free magnetic electrodes of magnetic tunnel junction devices.

In this paragraph, we address the possibility of stabilizing skyrmions in patterned disks by writing them from a saturated ground state, using the STT effect of a spin-polarized current injected perpendicularly across the disk. The geometry used for this micromagnetic simulation is represented in Figure 9. It is compatible with a magnetic tunnel junction (MTJ) element, either the individual or as the writing/reading part in a racetrack memory device. Within the hard–soft architecture of the MTJ, the pillar structure has the following parts: (1) a bottom ferromagnetic disk, constituted from the soft magnetic material in which the skyrmions will be written (free magnetic layer), (2) an insulating tunnel barrier, and (3) a top fixed (hard) magnetic layer acting as a spin polarizer for the current injected across the insulator. For the Mumax3 micromagnetic simulations we used the following geometry: 1 nm thick circular disks of 90 nm diameter for the free (bottom) layer and 20 nm diameter for the hard (top) polarizer, respectively. The fixed and spacer layer parameters in Mumax3 code, required for including the Slonczewski and Zhang-Li spin transfer torques effects [27] were set up as follows: the Slonczewski Λ parameter λ = 1, the electric current polarization Pol = 0.4–1, the Slonczewski secondary STT term ε’ = 0, the non-adiabaticity parameter of the Zhang-Li model ξ = 2, and the magnetization of the fixed layer was locked along the (001) perpendicular direction. The material parameters for the free layer in which the skyrmions will be written were chosen to be exactly those issued from the ab-initio calculations for the Au/Fe(5ML)/MgO system: K_u_ (PMA) = 1.91 × 10^+6^ J/m^3^ and D_ind_ (DMI) = 1.67 × 10*^−^*^3^ J/m^2^; M_s_ = 1714 × 10^+3^ A/m and α = 0.01 corresponding to the bulk Fe. In our simulations, we also considered as a variable parameter the voltage modulation of the anisotropy ( *ξ*_VCMA_) and the DMI for the bottom free layer. During the current pulse, the MTJ gets polarized and the electric field across the insulator was chosen to reduce the PMA and the DMI: K_u_ = Ku^ini^(1-ξ_VCMA_) and D_ind_ = D_ind_^ini^(1-ξ_VCMA_); the initial Ku^ini^ and DMI^ini^ values being restored after the pulse. The size of the unit cell of the micromagnetic grid was 1 × 1 × 1 nm.

**Figure 10:**
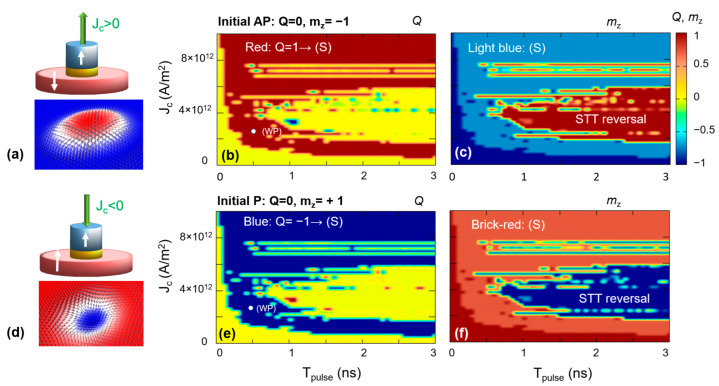
(**a**,**d**) Simulation geometry and final skyrmionic state magnetization vector field used in skyrmion writing by STT. T_pulse_-J_c_-Q and T_pulse_-J_c_-m_z_ phase diagrams, starting either from an initial antiparallel (AP) state (**b**) and (**c**) or parallel (**P**) state (**e**) and (**f**).

Within this framework, we have calculated various T_pulse_-J_c_-Q and T_pulse_-J_c_-m_z_ phase diagrams, starting either from an initial antiparallel (AP) or parallel (P) state of the fixed polarizer and the free layer; T_pulse_ is the length of the current pulse, J_c_ the density of the polarized electric current, Q the topological charge and m_z_ the normalized z component of the magnetization. The micromagnetic grid used is a unit cell of 1 × 1 × 1 nm and the diagrams are built from 10.000 relaxed dynamics. In Figure 10, we illustrate the skyrmion writing phase diagrams calculated considering a current polarization *p* = 0.8 and a voltage modulation of anisotropy and DMI, ξ = 0.1. Like in common experiments of magnetization switch by STT, as a function of the initial state, P or AP, the direction of the injected J_c_ for writing the skyrmions by STT must be reversed (see Figure 10a,d). The T_pulse_-J_c_-Q diagrams (Figure 10b,d), corresponding to injection from AP(P) initial states, illustrate the J_c_ and T_pulse_ parameter range allowing to write a skyrmion: either with a positive core polarization (Figure 10a), when STT writing from an initial AP saturated state or a negative core polarization (Figure 10d), when injecting from an initial P state. Analyzing the sign and the value of the topological charge of the skyrmionic states range of the T_pulse_-J_c_-Q diagrams: (Q = 1, red color in (b) or Q = –1, blue color in (e)), one can see that, depending on the initial AP or P state, the core polarization, and the chirality of the written skyrmion will be opposite. Moreover, the T_pulse_-J_c_-m_z_ phase diagrams (Figure 10c,d) bring complementary information, indistinguishable from the T_pulse_-J_c_-Q diagrams. They allow us to discriminate the parameter range J_c_ –T_pulse_ leading to the full reversal of the free-layer disk by STT, the STT writing experiments in STT-RAMs are the same.

**Figure 11:**
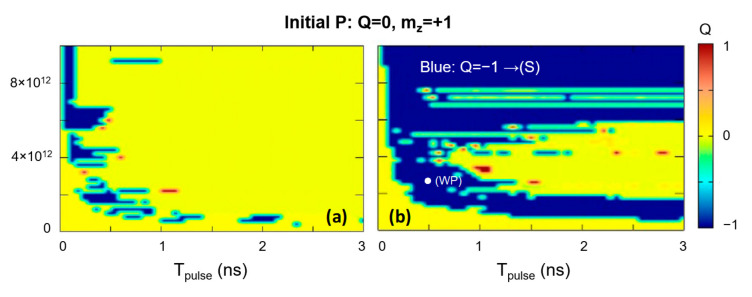
T_pulse_-J_c_-Q phase diagrams for a 60 nm disk (**a**) and 90 nm disk (**b**) starting from an initial parallel (**P**) state.

Similar phase diagrams, calculated for disks with various sizes, illustrate that the efficiency of the STT writing increases with increasing the disk diameter (see Figure 11). The J_c_ and T_pulse_ window for the (S) writing by STT is significantly larger for the 90 nm disks as compared to 60 nm ones.

**Figure 12:**
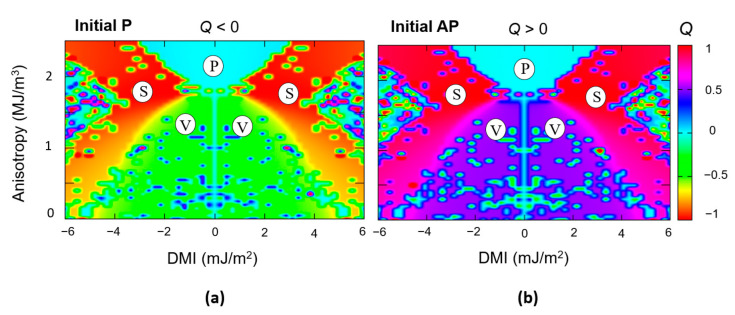
*DMI-PMA-Q* phase diagrams corresponding to simulation experiments started either from an initial P state (**a**) or an initial AP state (**b**). The parameters of the current pulse used in the simulation were: *T_pulse_* = 0.5 ns and *J_c_* = −3 × 10^12^ A/m^2^ or *J_c_* = + 3 × 10^12^ A/m^2^, depending on the initial P or AP state (WP in Figure 22a,d). The sign of the written skyrmion topological charge is opposite in the two situations.

Based on the information brought by the T_pulse_-J_c_-Q and T_pulse_-J_c_-m_z_ phase diagrams, we have chosen a specific point whose parameters (J_c_, T_pulse_) are suitable for writing a skyrmionic state by STT (white point designed by (WP) in Figure 10b,e). Corresponding to these (J_c_, T_pulse_) values that were kept fixed, we have calculated new phase diagrams in which the PMA and the DMI have been varied to potentially cover a broader range corresponding to other skyrmionic materials. The diagrams illustrated in Figure 12, correspond to a 90 nm diameter circular nanodisk, patterned from a 1 nm thick ferromagnetic film, and are built from 10.000 relaxed dynamics in which we chose as variables the anisotropy and the asymmetric exchange (DMI) and fixed the other magnetic parameters: the saturation magnetization M_s_ = 1714 kA/m; the exchange stiffness A_ex_ = 2.1 × 10^+11^ J/m; the Gilbert damping constant α = 0.01, which are typical for a Fe thin film that can enter in a X/Fe(5ML)/MgO multilayer stack. The color scale represents the value of the topological charge: $Q=\frac{1}{4\pi }\int d^{2}x\;\vec{m}\cdot \left(\frac{d\vec{m}}{dx}\times \frac{d\vec{m}}{dy}\right)$ used to classify the micromagnetic configuration of the final relaxed chiral ground states. In our simulations, we start from an initial saturated P or AP (with respect to the hard reference layer) state, and after a current pulse (J_c_, T_pulse_) we cut the pulse and determine the final relaxed micromagnetic configuration of the disk. 

These diagrams, illustrated in Figure 12, show that the stabilization of a skyrmionic state occurs in an optimum window of DMI; the larger the DMI, the wider the PMA window will be in which skyrmionic states can be injected by a current. Above a certain DMI value, the skyrmionic states become unstable in a PMA range increasing with increasing DMI, and other complex chiral structures will be stabilized. When writing the skyrmion by STT using a current pulse, above a certain DMI value the skyrmionic final states become also unfavorable. After the current pulse, the system will relax towards a vortex, a perpendicularly saturated or a complex chiral final state. This will happen within a PMA window whose width increases with the increase in the DMI magnitude.

To get a deeper understanding of the mechanisms and magnetic free-energy contributions responsible on the stabilization and the stability of skyrmionic states, we extended our study to phase diagrams of ground states in nanodisks with different sizes and shapes. 

#### 3.2.2. Skyrmionic Ground States in Patterned Nanodisks

**Figure 13:**
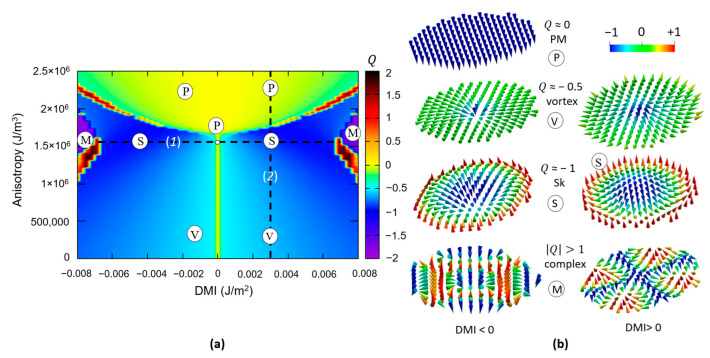
(**a**) Ground state diagram *DMI-PMA-Q* (90 nm circular disk) in which the micromagnetic states are classified after their topological charge *Q*. (**b**) Micromagnetic configuration in vector field glyph representation corresponding to chosen zones from the phase diagram: (P) = perpendicularly magnetized (PM) states, (V) = magnetic vortex states (*Q* ≈ −0.5), (S) = magnetic skyrmionic states (*Q* ≈ −1), (M) = complex chiral magnetic states with $\left|Q\right|$ >1 for different vorticity chiral structures, determined by the sign of the DMI.

**Figure 14:**
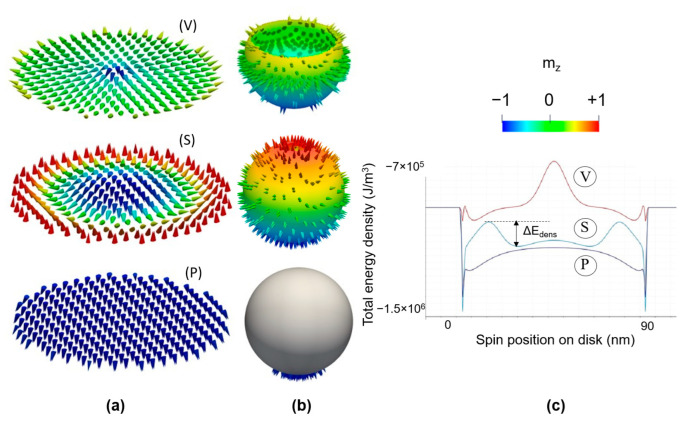
Micromagnetic configuration in vector field glyph representation (**a**) and (**b**) projections on the Bloch sphere of the corresponding magnetization vector for: (V) = magnetic vortex states (*Q* ≈ – 0.5), (S) = magnetic skyrmionic states (*Q* ≈ −1) and (P) = perpendicularly magnetized (PM) states; (**c**) Line profile of the total energy density of the spins within the ferromagnetic disk.

The ground state of the magnetic nanostructure has been obtained by relaxing the LLG magnetization dynamics, starting from an initial saturated state, chosen with the magnetization aligned along the –Oz = (00-1) direction. Figure 13 illustrates such a diagram, corresponding to a 90 nm diameter circular nanodisk, patterned from a 1 nm thick ferromagnetic film. The parameters used in these simulations are the same as those used for generating the diagram from Figure 12. Starting from this diagram, we analyzed the evolution of the ground state micromagnetic configurations when fixing a parameter and varying the other (e.g., PMA = 1.5 × 10^+6^ J/m^3^, DMI variable–line (1)) and DMI = 3 × 10*^−^*^3^ J/m^2^, variable PMA–line (2). The resulting magnetic features are displayed in Figure 13 and Figure 14 and the following conclusions have been drawn:

- At a fixed DMI, at small PMA values, the ground state is vortex-like, with Q ≈ −0.5; when the PMA increases, within a certain range whose extension increases with the value of the DMI, the ground state becomes skyrmionic Q ≈ −1; above a threshold maximum value of the PMA, the ground state will be a saturated monodomain.
- Within the skyrmionic window, the skyrmion’s diameter progressively decreases with the PMA increase. The narrow transition from a skyrminonic to a saturated state implies some complex chiral states with $\left|Q\right|>1$.
- The skyrmionic states are stabilized by larger values of DMI but, above a critical value, at fixed PMA, large DMI will favour $\left|Q\right|>1$ configurations within a PMA window increasing with the increase in the DMI.
- In conclusion, an optimum window of PMA and DMI values is required to stabilize a skyrmionic ground state.

The magnetization vector field projection on the Bloch unit sphere of the Néel chiral structures (“hedgehog”) illustrates the correlation between the value of the topological charge Q and the “population” of the Bloch sphere by spins. For a vortex state, half of the Bloch sphere is populated, a skyrmionic magnetization vector field populates the entire sphere and a saturated state populates either the (0;0;−1) or the (0;0;1) poles of the unit sphere $m_{x}^{2}+m_{y}^{2}+m_{z}^{2}=1$.

**Figure 15:**
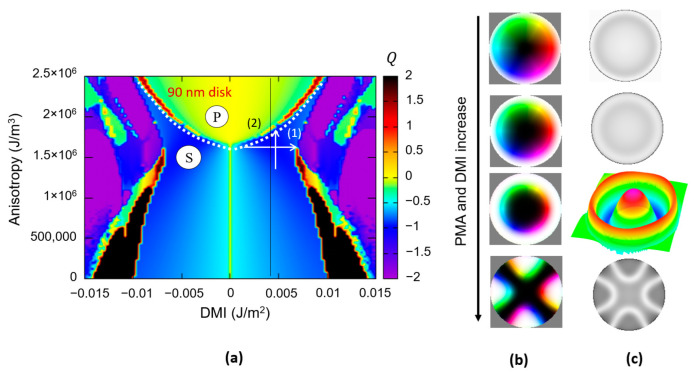
(**a**) Ground state diagram *DMI-PMA-Q* (90 nm circular disk) with two analysis paths: (1): fixed PMA = 1.72 × 10^+6^ J/m^3^ and (2): fixed DMI = 5 mJ/m^2^. (**b**) Micromagnetic chiral ground states and (**c**) Total energy density landscapes evolution when the PMA and the DMI increase along the paths (1) and (2). In (**a**), the white dotted lines are guiding lines for the threshold from (S) to (P) state, and the black vertical line corresponds to the analysis path used in Figure 13: fixed DMI *D_ind_* = 4 mJ/m^2^ and variable PMA *K_u_* = 0−2.5 × 10^+6^ J/m^3^.

The total energy of the final states is different. The analysis of a line profile in the total magnetic energy density of the disk demonstrates that the vortex state is less energetically favorable for the inner spins and that the skyrmionic state is a topologically stabilized configuration (Figure 15c).

Here, for the spins in the inner skyrmionic core, an energy barrier $\Delta E_{dens}$ prevents them to rotate towards the opposite orientation of the edge spins. The height of this barrier is a complex energetical competition issue between the favorable DMI and PMA contributions and unfavorable magnetostatic and direct exchange. For room temperature skyrmion stability, the barrier energy has to be larger than the thermal energy $k_{B}T$, $k_{B}$ being the Boltzmann constant and T the value of the absolute temperature (more details can be found in Appendix A).

Within the skyrmionic range of the phase diagram (Figure 15) the increase in either PMA or DMI will lead to the shrinkage of the skyrmionic core. 

When the DMI increases at fixed PMI (path (1)), the skyrmonic size decreases, but above a critical DMI threshold complex chiral structures with Q > 1 become stable. In the case of the PMA increase at fixed DMI (path (2)), above a critical threshold, the core shrinks to collapse into a saturated state with an opposite magnetization orientation with respect to the one of the initial skyrmion core. As illustrated in the next paragraph, this behaviour can be used for the core-reversal strategy under external applied electric field. The transition from (S) to (P) state goes through some (M) metastable states in a very narrow PMA window. 

**Figure 16:**
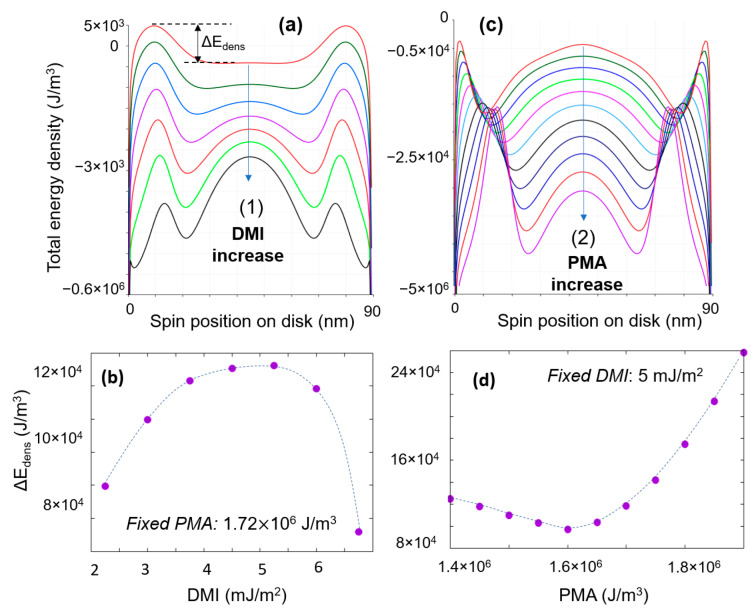
Total energy density line profile evolution with DMI and PMA for: (**a**) DMI increase at fixed PMA (along the line (1), Figure 15a) and (**b**) PMI increase at fixed DMI (line (2), Figure 15a). Evolution of the energy barrier $\Delta E_{dens}$ with the increase in the DMI (**c**) and PMA (**d**).

The variation of the energy density barrier $\Delta E_{dens}$ separating the skyrmion core spins and the oppositely oriented edge ones has a non-monotonous evolution with DMI and PMA, as illustrated in Figure 16. In both situations, an optimum window exists for stable skyrmionic states (Figure 16b,c): complex chiral structures with Q > 0 or saturated state (Q = 0) appearing after some up-threshold DMI and PMA values, respectively. A larger PMA implicates a confinement of the skyrmion core in a smaller region. This would require a larger DMI for twisting the spins against the direct exchange interaction. The threshold from (S) to (P) is roughly showing a parabolic increase with the DMI (Figure 15a) dotted white line. 

**Figure 17:**
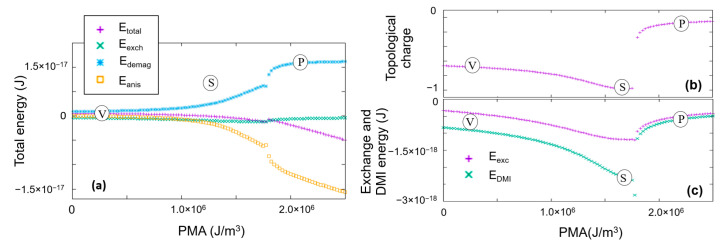
Variation with the anisotropy at fixed DMI for: (**a**) different contributions to the magnetic free energy; here, the exchange energy includes both the DMI and the direct exchange (**b**) topological charge (**c**) total exchange and only the DMI contributions energies.

A deeper insight into the skyrmion stability, with respect to the increase in the anisotropy, can be obtained by analyzing the corresponding variation of different contributions to the magnetic free energy (Figure 17). The analysis has been performed along a path, indicated by a solid black vertical line in Figure 15, corresponding to a fixed value of DMI (D_ind_ = 4 mJ/m^2^) and variable PMA (K_u_ = 0–2.5 × 10^+6^ J/m^3^). We see that the skyrmion stability is a complex issue, being determined by the balance between the PMA, the DMI, the direct exchange and the demagnetizing (magnetostatic) energies. When the PMA increases during the evolution from a vortex state to a skyrmionic one, the total energy decreases. However, this evolution costs more and more in demagnetizing energy which is continuously increasing. At a certain critical value of PMA, an abrupt transition into a saturated perpendicular state will stop the increase in the magnetostatic energy and will accommodate both the anisotropy and the direct exchange energies, in detriment to the DMI energy (the abrupt transition from (S) to (P) is also illustrated in Figure 17).

**Figure 18:**
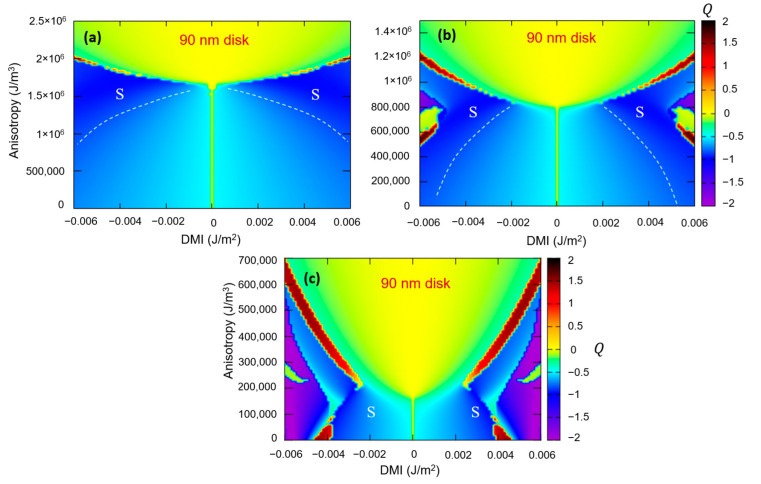
The influence of the saturation magnetization on the DMI-PMA-Q ground state phase diagram (**a**) Fe type ferromagnetic material with *M_s_* = 1714 kA/m; *A_ex_* = 2.1 × 10^−11^ J/m (**b**) Co type material with *M_s_* = 1200 kA/m; *A_ex_* = 1.8 × 10^−11^ J/m and (**c**) CoFeB type material with *M_s_* = 580 kA/m; *A_ex_* = 1.5 × 10^−11^ J/m.

The magnetostatic demagnetizing energy is mainly influenced by two parameters: the saturation magnetization M_s_ and the size and the shape of the magnetic disks. Both have a direct impact on the DMI-PMA-Q ground state phase diagram, and, implicitly, on the skyrmion stabilization parameters window. This is illustrated by Figure 18 which depicts phase diagrams corresponding to three materials commonly used in spintronic devices, with different saturation magnetization values: Fe (M_s_ = 1714 kA/m; A_ex_ = 2.1 × 10*^−^*^11^ J/m), Co (M_s_ = 1200 kA/m; A_ex_ = 1.8 × 10*^−^*^11^ J/m) and CoFeB (M_s_ = 580 kA/m; A_ex_ = 1.5 × 10*^−^*^11^ J/m). One can see that the decrease in M_s_ and A_ex_ promotes the skyrmion formation at larger DMI and PMA values and favor chiral structures with $\left|Q\right|>1$. From our simulations, we deduce that materials with a large M_s_ value would promote skyrmion stabilization at lower DMI but, for that to happen, larger PMA values would be required. Moreover, large M_s_ values are detrimental for complex chiral structures with $\left|Q\right|>1$.

**Figure 19:**
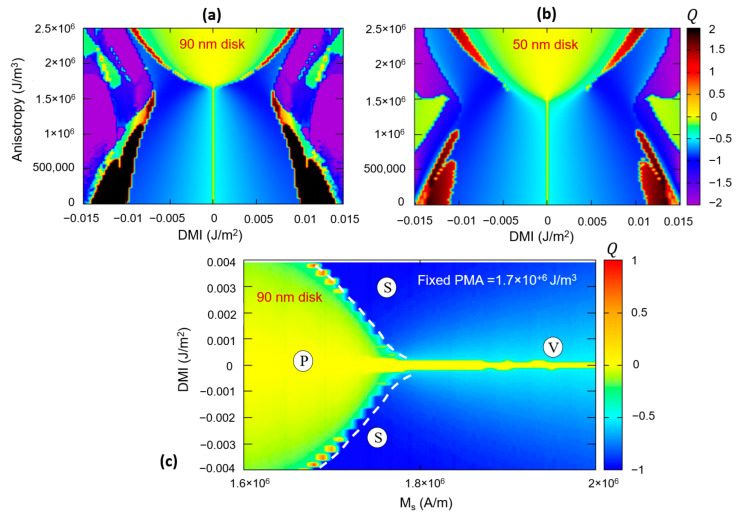
The influence of the ferromagnetic disk size on the *DMI-PMA-Q* ground state phase diagram. (**a**) 90 nm diameter disk (**b**) 50 nm diameter disk (**c**) *M_s_-DMI-Q* phase diagram indicating the maximum DMI required to promote skyrmionics (S) state instead of vortex (V) for a given M_s_.

The influence of the size of the magnetic disks is illustrated in Figure 19, in which we represented calculated DMI-PMA-Q phase diagrams for 90 nm and 50 nm disks patterned from Fe-type materials with M_s_ = 1714 kA/m; A_ex_ = 2.1 × 10^−11^ J/m. One can clearly observe that the smaller ferromagnetic disks would need a larger DMI to promote skyrmionic states (because the core should be confined within a smaller area and spins are swirling within a narrow length–scale). A direct consequence would be the necessity to fabricate materials and/or profit from additive effects in complex multilayered architectures to gain access to the large DMI values necessary to host nanometric size skyrmions in nanometric disks, as required for high-density storage or qubit applications. As illustrated in Figure 19c, this issue can be partially overcome by using materials with a larger M_s_: the minimum value of DMI for skyrmionics ground state stabilization decreases with the increase in M_s_.

**Figure 20:**
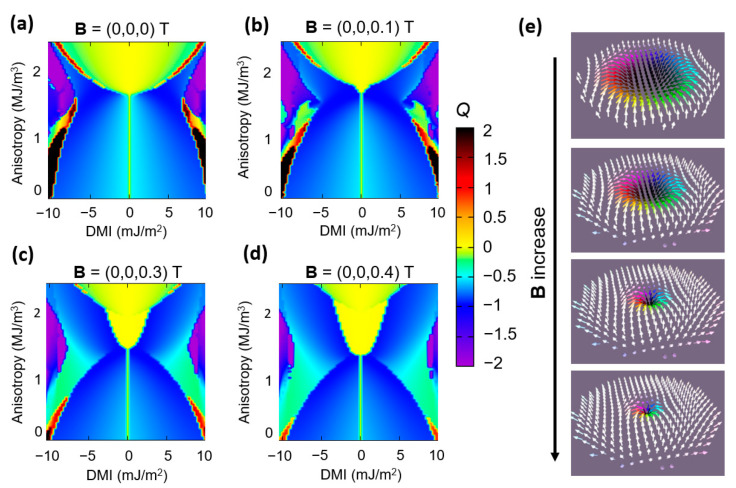
The influence of the magnetic field applied along the +Oz axis on the *DMI-PMA-Q* ground state phase diagram for a 90 nm disk. (**a**) B = 0 T (**b**) B = 0.1 T (**c**) B = 0.3 T (**d**) B = 0.4 T. (**e**) The variation of size of the skyrmion core with increasing magnetic field. The images correspond to a point (DMI = 5 mJ/m^2^, K_u_ = 1.7 × 10^+6^ J/m^3^). The calculation has been performed for a 90 nm disk with the following fixed parameters: *M*_s_ = 1714 kA/m, *A*_ex_ = 2.1 × 10^+11^ J/m; the Gilbert damping constant α = 0.01.

From all the DMI-PMA-Q diagrams (Figure 13a, Figure 15a, Figure 18, Figure 19 and Figure 20) and from the variation of the value of the topological charge Q with the increase in the anisotropy (Figure 17b), one can identify the manifestation of the topological protection of skyrmions. The transition between the skyrmionic domain ($\left|Q\right|\approx 1$) towards the uniform magnetization one ($\left|Q\right|\approx 1$) is always abrupt (e.g., when enhancing the anisotropy). 

Another investigation that we performed concerned the influence of a perpendicularly applied magnetic field on the magnetic nano-disk ground states. This aspect is illustrated in Figure 20. The first visible/sizeable effect concerns the vanishing of complex chiral states with positive topological charge $\left|Q\right|>1$ in favor of vortex configurations. Then, the parameter window for stabilizing skyrmions is pushed towards smaller DMI and PMA values and enlarged for the higher PMA range. Moreover, the size of the skyrmion shrinks with increasing the magnetic field (as already shown by Moreau-Luchaire et al. in [9]). This could be an interesting issue for applications from both materials and skyrmion manipulation points of view.

The studies presented in this paragraph relative to the stabilization of different types of chiral ground states illustrate the importance of material engineering, e.g., proper choice and control of M_s_, A_ex_, PMA, DMI, to be able to fit within the parameter range required for skyrmion stabilization, in view of any skyrmionic application. These concepts are not only valid for nanostructures constituted from single magnetic layers but can be further extrapolated to more complex heterostructures. 

**Figure 21:**
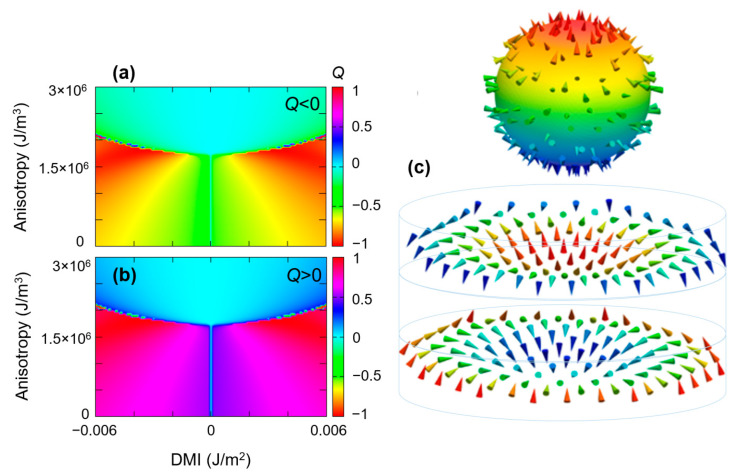
Antiferromagnetic configuration *DMI-PMA-Q* ground state phase diagram, for the top (**a**) and bottom (**b**) disks antiferromagnetically coupled (e.g., in a Synthetic antiferromagnet) (**c**) Representations of the magnetization vector fields in the two AF coupled layers illustrating opposite spins and sign of the topological charge (chirality). This configuration corresponds to a skyrmionic AF ground state corresponding to *PMA* = 1.74 × 10^+6^ J/m^3^ and *DMI* = −4 × 10^−3^ J/m^2^.

**Figure 22:**
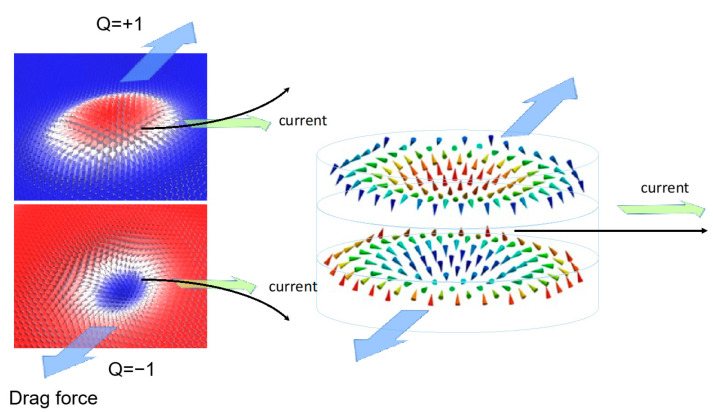
Illustration of correction of the “Magnus” drift due to skyrmionics Hall effect in AF coupled skyrmions.

Indeed, in Figure 21, we present an example of a DMI-PMA-Q ground state phase diagram corresponding to an antiferromagnetic (AF) configuration. The diagram looks very similar to the one corresponding to a single layer, the chiral structures (e.g., skyrmions) in the two layers are antiferromagnetically mirrored and have opposite signs of the topological charge (Figure 21c). Such kinds of skyrmions could be stabilized in synthetic antiferromagnetic multilayers. They would have the advantage that the two FM layers which can be finely tuned are joined together for an increased thermal stability and improved dynamical behavior of the Skyrmion, e.g., correction of the “Magnus” drift or skyrmionic spin Hall effect (Figure 22), observed if single skyrmions move in racetracks [31]. Moreover, as recently suggested, such kind of AF-coupled skyrmions could be used as coupled qubits [19]. 

**Figure 23:**
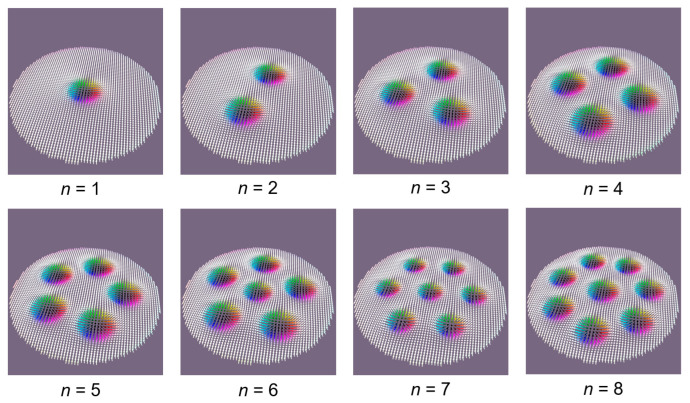
Multi-skyrmionic ground states obtained in 250 nm diameters disks.

At the end of this subsection, we would like to illustrate that multi-skyrmionic ground states can be also generated in nano-disks by properly tuning the M_s_, PMA, and DMI parameters (Figure 23). The simulations presented in this figure are performed considering a 250 nm diameter disk, large enough to be able to accommodate multiple skyrmions within a PMA-DMI window in the range of phase diagram parameters of Figure 14c; the simulations being for a CoFeB type material with M_s_ = 580 kA/m; A_ex_ = 1.5 × 10*^−^*^11^ J/m.

The possibility to generate such kind of multiple skyrmionic states by PMA and DMI tuning (e.g., by electric field gating), is particularly important for memristor functions, e.g., in a racetrack, where the resistance read by a magnetic tunnel junction depends on the number of skyrmions displaced by the current [18].

#### 3.2.3. Skyrmionic Nano-Oscillators

Once the skyrmions are generated, an important issue for a different type of applications is their manipulation in a different type of device.

**Figure 24:**
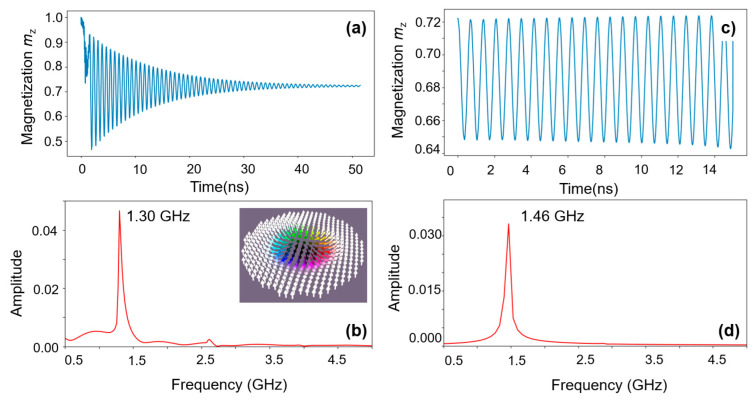
(**a**) Time dynamics of the magnetization component m_z_ during the STT writing event. (**b**) Fast Fourier Transform (FFT) spectrum for the damped oscillations illustrated in (**a**). (**c**) Time dynamics of the skyrmionic STT oscillator for which the amplitude is kept constant by the anti-damping torque provided by STT of the spin-polarized current. (**d**) FFT spectrum corresponding to the skyrmionic STT oscillator.

The *T_pulse_-J_c_-Q* and *T_pulse_-J_c_-m_z_* phase diagrams illustrate that for writing a skyrmion by STT, a spin-polarized current pulse must be applied, with properly chosen *J*_c_ and *T*_pulse_. After cutting the pulse, the skyrmionic nucleated pattern will have a damped oscillation whose size and shape is evanescently converging towards the final diameter imposed by the magnetic material parameters (DMI, PMA, M_s_) and the patterned disk geometry. However, as if for a mechanical damped oscillator, we would supply energy to compensate the damping losses, e.g., by acting with an external driving torque provided by a spin polarized current that competes with the damping torque, the skyrmion can be driven in a steady self-sustained oscillation regime: in which the size of the skyrmion is cyclically increasing and decreasing (so-called “breathing” regime). In Figure 24, we illustrate micromagnetically simulated magnetization dynamics feature for the writing (damped oscillations from pulse nucleated pattern towards the final skyrmionic state) and the steady oscillation regime. They concern a skyrmion generated in a 90 nm diameter nano-disk, patterned from an Au/Fe(5ML)/MgO multilayer system with the magnetic parameters extracted from ab-initio calculations: *K*_u_ (PMA) = 1.91 × 10^+6^ J/m^3^ and *D*_ind_ (DMI) = 1.67 × 10^−3^ J/m^2^; *M*_s_ = 1714 × 10^+3^ A/m and *α* = 0.01 are corresponding to bulk Fe.

The conditions used for writing and sustaining the oscillations were: *J*_c_ = 1 × 10^+12^ A/m^2^ and T_pulse_ = 1.5 ns for writing from an initial P state of the MTJ nanopillar, then *J*_c_ = −1.5 × 10^+12^ A/m^2^ for STT driving torque driven oscillations. The spin-polarized current *J*_c_ injected into the nanopillar has a double effect: the damping-like component will provide the negative torque for sustaining the steady oscillations and the field-like component will modulate the frequency of oscillation determining a strongly nonlinear variation with the current density *J*_c_. For maintaining the steady oscillation regime of the skyrmionic nano-oscillator, *J*_c_ must be adjusted in a proper range. If it is too small, the damping will not be entirely compensated, and the oscillations will gradually vanish. *J*_c_ values which are too large will provide anti-damping torques which are also too large. The skyrmion core will increase up to the limit of the disk size, and collapse in a stable saturated state. The skyrmionic nano-oscillators [32] gained a lot of interest in recent years. Their wide window of operating frequency, in the range of the microwaves (1–100 GHz), tunable by external stimuli (magnetic fields, STT of spin-polarized currents), open interesting application perspectives as nanoscale electric oscillators, and sensitive magnetic field sensors [33]. Their strongly nonlinear dynamics can be used in studies of chaotic phenomena [34] and for neuro-inspired devices suitable for neuromorphic applications, e.g., pattern recognition [35]. 

#### 3.2.4. Skyrmion Manipulation by Electric Fields

**Figure 25:**
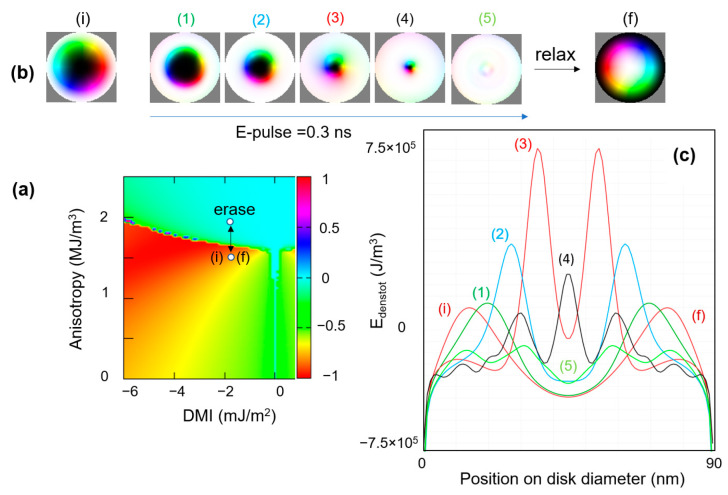
(**a**) Zoom on the *DMI-PMA-Q* phase diagram. (**b**) Micromagnetic states of skyrmion: (i) = initial state, (1)–(5) intermediate states during the E-field pulse, (f) final state, after cutting the pulse. (**c**) Line profiles of the total magnetic energy density in the disk.

Once the skyrmion is generated, the next issue is to manipulate them in skillfully designed classic (storage, logics), neuromorphic and quantum devices. The strategy of skyrmion manipulation by electric fields is one of the most energetically efficient options. It is based on the electric field control of the perpendicular magnetic anisotropy [36]. In Figure 25a, we zoom on a skyrmionic zone within a ground states *DMI-PMA-Q* diagram. We set an initial skyrmionic state (i), described by *PMA* = 1.675 × 10^+6^ J/m^3^ and *DMI* = 2.4 × 10^−3^ J/m^2^, chosen to be in the proximity of the transition zone between the skyrmionic (S) and the saturated (P) states. This initial skyrmion, being nucleated from a saturated state along −*Oz* m = (0,0,−1), has a negative core polarization, the other parameters used in the simulation are *M_s_* = 1714 × 10^+3^ A/m, *A_ex_* = 2.1 × 10^−11^ J/m and *α* = 0.01 (Fe). 

Afterwards, we simulate the application of an electric field pulse with a properly adjusted delay (here 0.3 ns) and with an orientation providing an enhancement of the PMA. The E-field increase in PMA would lead to the progressive decrease in the skyrmion diameter, up to the complete erasure (Figure 25b states (1)–(5)). After cutting down the pulse, the anisotropy is restored to its initial value and a new skyrmionic state with an opposite core orientation will be relaxed as the ground state. In Figure 25c, we illustrate the line profiles of the total magnetic energy density corresponding to the states depicted in Figure 25a. The core polarization always corresponds to the orientation of the disk magnetization in the saturated state before relaxation. When erasing a skyrmion with negative polarization by core shrinkage, we end with an up-saturated state and then, the new relaxed skyrmionic ground state will have a positive core polarization, and vice-versa. 

**Figure 26:**
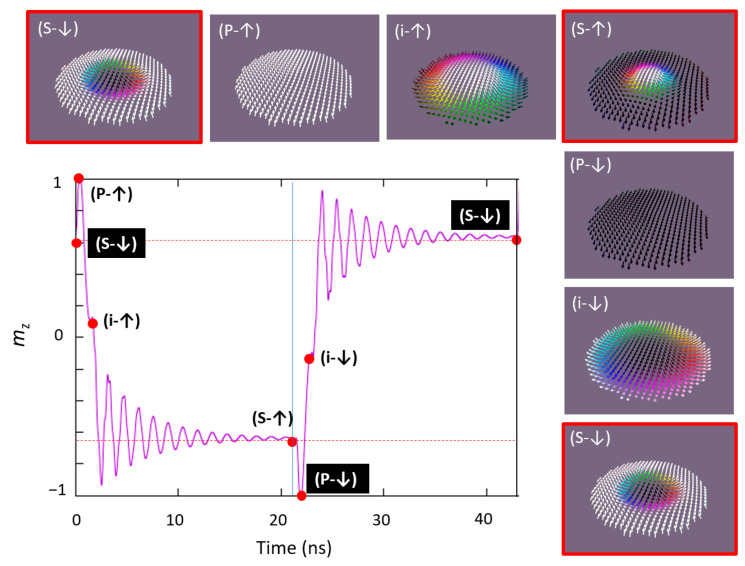
Magnetization dynamics of the *m*_z_ component during two successive cycles of skyrmionic core reversal by electric field. Corresponding micromagnetic configurations of some representative magnetic states: skyrmion with up(down) core orientation [S- ↑(↓)], saturated perpendicular up(down) [P-↑(↓)], intermediate up(down) states [i-↑(↓)], are represented.

Following this strategy, as illustrated in Figure 26, the skyrmionic core can be cyclically reversed from up to down orientation. These results demonstrate the capability of the electric field control of a binary (0)/(1) information that could be encoded in the skyrmion core magnetization, e.g., if the magnetic disk containing the skyrmion is the free magnetic layer of a magnetic tunnel junction whose resistance depends on the skyrmionic core polarization. 

**Figure 27:**
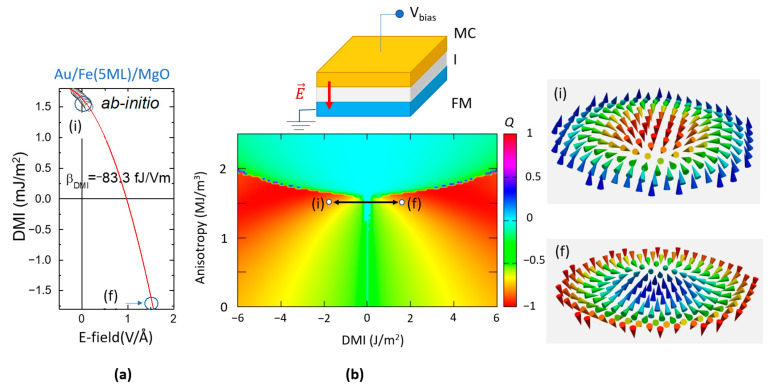
(**a**) Ab-initio calculated DMI variation with the electric field. The variation rate is described by the parameter $\beta =\frac{\Delta DMI}{\Delta E-field}$ (fJ/Vm). (**b**) *DMI-PMA-Q* phase diagram in which we indicated the path between the initial (i) and the final (f) states showing opposite core polarization and chirality. Top insert: sketch of E-field biasing geometry, the electric field at the surface of the ferromagnetic layer (FM) is applied across an insulator (I) using a top metallic contact electrode (MC).

On the other hand, an interesting issue would be related to the possibility to switch the chirality and the core polarization of a skyrmion, simultaneously. Our simulations indicate that this can be performed by exploiting the electric field modulation of the DMI. From ab-initio calculations, we can determine the rate of the electric field variation of the DMI, as illustrated in Figure 27a. We fix an initial state (i) for a value of DMI allowing a ground state skyrmionic stabilization (blue open circle). Then, from the theoretical DMI vs E-field curve, the value (and the orientation) of the electric field required to reverse and obtain a positive DMI value, providing a new (f) skyrmionic ground state with opposite chirality, can be extrapolated. Following the path between (i) and (f) the simulated magnetization dynamics show that the initial and the final skyrmion have opposite core polarization and chirality (as in the case of antiferromagnetically mirroring). This possibility for core and chirality reversal can be useful in storage or logic devices where, by E-field gating one can change the sign of the skyrmionic Hall effect and the direction of the skyrmionic drift.

However, as illustrated in Figure 27a, if only intrinsic effects are responsible for the DMI variation with the electric field, a quite large gating voltage is required. From a practical point of view, this could bring limitations due to the dielectric breakdown issues of the insulator used for the voltage gating (experimental geometry depicted in the top insert of Figure 27). Therefore, beyond optimizing the quality of the dielectrics used for the voltage gating, a major challenge for the implementation of the DMI sign reversal in skyrmionic applications would be to design and elaborate materials and related multilayered configurations in which the skyrmionic states can be stabilized at low DMI and PMA values.

## 4. Discussion

### 4.1. Multiscale Modeling of Skyrmionic Nanomaterials

**Figure 28:**
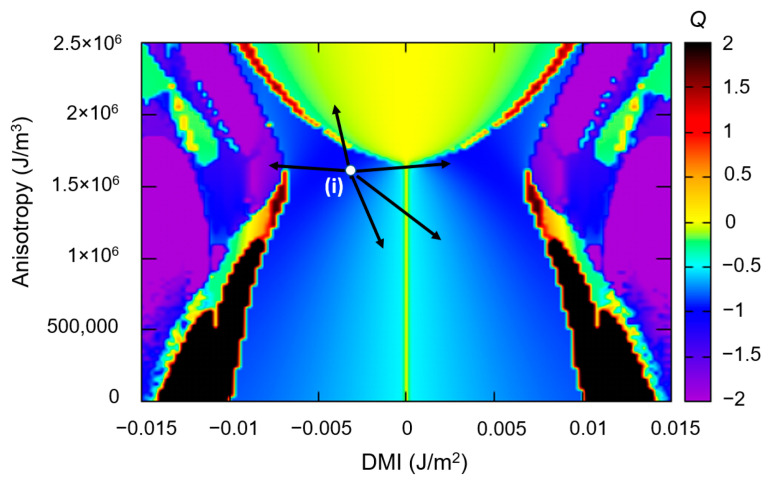
Possible paths of “motion” within a *DMI-PMA-Q* diagram, starting from an initial point (i), e.g., by electric field gating. The magnetic parameters used in *Mumax3* are those for the diagram illustrated in Figure 13 and Figure 15.

The analysis illustrated in Figure 27 demonstrates that a multiscale strategy, combining ab-initio and micromagnetic modeling, can be particularly useful for theoretically investigating skyrmionic or other topological spin texture materials. In our case, the ab-initio analysis provided a fundamental insight into the PMA and DMI mechanisms and quantified their electric field control capability in various ab-initio materials and multilayer architectures experimentally conceivable by standard deposition techniques (i.e., Molecular Beam Epitaxy, Sputtering). As sketched in Figure 28, this would indicate the theoretically possible paths of “motion” within a *DMI-PMA-Q* diagram to access and manipulate the available different chiral magnetic states. Extrapolated beyond this example, in the first stage, the ab-initio analysis could be employed to get insight into the anatomy of intrinsic properties and phenomena allowing to theoretically design materials or multilayer configurations with optimum magnetic properties. Then, in the next stage, their mesoscopic static and dynamic magnetic properties can be further tested/simulated using micromagnetic tools. The multiscale modeling can be also done the other way around. From micromagnetic extended phase diagrams one can identify the range of optimum parameters for the envisaged magnetic configuration and nanoscopic property. Then, ab-initio calculations can be used to design the magnetic material or complex nanostructure that would provide those properties. If such a kind of multiscale analysis precedes the experimental elaboration of complex nanomaterials, the net advantage would be the reduction in the experimental efforts, costs, and time consumption. 

### 4.2. Experimental Issues on Magnetic Skyrmionic Nano-Materials 

**Figure 29:**
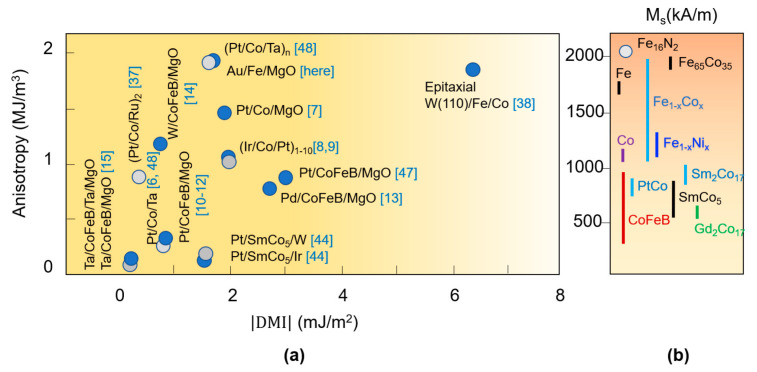
Selected data from the literature concerning (**a**) PMA and DMI and (**b**) saturation magnetization M_s_ values, fitted within the parameters range used in our micromagnetic calculations. When only the surface contribution to the PMA (*K_s_*) was available, the anisotropy *K_u_* has been calculated considering a ferromagnetic thickness layer of t = 1 nm: *K_u_* = *K_s_*/t; when the anisotropy field $B_{k}=\mu_{o}H_{k}$ (T) was available for a layer with given *M_s_* (A/m), *K_u_* (J/m^3^) was calculated as $K_{u}=M_{s}H_{k}/2\;$[47,48].

Based on the predictive theoretical calculations, we got insight into the magnetic properties of selected materials and layered architectures and placed them on the different types of phase diagrams describing different magnetic chiral states. Then, we illustrated the possibility to manipulate the magnetic skyrmionic states for various applications. The last issue that we would like to discuss is about some currently available magnetic materials and corresponding layered thin film architectures that could accommodate the DMI, PMA and *M_s_* parameter range of our calculated phase diagrams. A selection with recent data available from the literature, concerning selected values of the saturation magnetization, DMI and PMA is illustrated by Figure 29 (the corresponding references are indicated). Concerning the saturation magnetization of a ferromagnetic layer –Figure 29b–considering its ultrathin thickness range, the surface contributions will be significant. Therefore, the value of *M_s_* depends on the thin film thickness and the chemical nature of the top (*X*) and bottom (Y) interface layers *X*/FM/Y. From Figure 29a, one can see that the anisotropy range is well covered by available experimental systems. They not only provide a wide range of surface anisotropies, but one also has the possibility to tune the value of *K*_u_ by the thickness of the ferromagnetic layer: *K_u_ = K_s_/t*. On the other hand, the currently available DMI range, for the most part of the studied systems, is beyond 3 mJ/m^2^. As mentioned, the DMI can be enhanced by additive effects [9] or skillful engineering of multilayered sequences, use of synthetic antiferromagnets [37], etc. Indeed, recently, in the epitaxial W(110)/Fe/Co bilayer system a giant DMI, of 6.55 J/m^2^ obtained by quantum engineering of the lattice symmetry has been demonstrated [38]. On the other hand, in this paper we show that ab-initio and micromagnetic complementary tools can be successfully used to calculate the magnetic properties of multilayer skyrmionic materials, the predicted DMI and PMA values being similar with those measured in experimental stacks. Moreover, we theoretically indicate a path for enhancing the DMI, the PMA and their response to external electric fields by the skillful engineering of interfaces: a monolayer of Pt inserted at the top Fe/MgO would enhance the PMA by almost an order of magnitude, from 1.91 to 15.77 MJ/m^3^, the DMI from 1.67 to 2.27 mJ/m^2^ and their response to an external electric field roughly by a factor of 2.

However, the yet existing experimental “gap”, opening roughly above 3 mJ/m^2^, shows that there is still plenty of space for theoretical and experimental research concerning the topological spin texture materials. A promising issue would be to combine interfacial and bulk DMI mechanisms requiring magnetic materials with intrinsic large spin-orbit interactions. Rare-earth transition metal alloys are more and more considered promising candidates for small skyrmions and ultrafast chiral spin texture dynamics applications [39]. Within this emerging topic, skyrmionic bubbles have been recently obtained [40] in Rare Earth (RE)-based REMn_2_Ge_2_ (RE = Ce, Pr, Nd) magnets, in a wide temperature range (220–320 K). Lattices of magnetic skyrmions have been observed in centrosymmetric rare earth compounds, such as Gd_2_PdSi_3_ and GdRu_2_Si_2_ [41]. Spontaneous topological magnetic transitions were identified in NdCo_5_ RE magnets [42]. Compact ferrimagnetic skyrmions were observed in DyCo_3_ films [43] and interfacial chiral magnetism and isolated skyrmions have been demonstrated in SmCo_5_-based magnetic multilayers featuring perpendicular magnetic anisotropy [44].

The topological chiral textures’ magnetic material spectrum is not only restricted to metals but also opened to insulating magnetic oxides. Recently, it was demonstrated that the DMI can be significantly controlled by rare earth orbital magnetism and strain engineering in insulating magnetic oxides [45]. Even the paradigm that skyrmion occurrence is strictly related to the lack of symmetry centers in crystals or interfaces has been newly invalidated by showing that skyrmions can be created in materials having centers of symmetry but possessing geometric frustration [46]. 

Within these complex and challenging problematics, multiscale studies combining theoretical and experimental investigations could complete the missing elements in the puzzle and answer the complex applicative issues.

## 5. Conclusions

Within a multiple-scale modeling framework, we addressed the problems of magnetic skyrmions. On specially designed multilayer stacks, ab-initio calculations provided insight into characteristic magnetic quantities of multi-layered heterostructures: the saturation magnetization, the anisotropy, the DMI and their response to a gating electric field. Using as input these results, micromagnetic calculations solving the Landau–Lifshitz–Gilbert dynamics, allowed us to identify critical regimes for writing skyrmions in patterned nanopillars. Extended phase diagrams, in which the magnetic textures were classified according to their topological charge, allowed us to identify the suitable range of magnetic anisotropy, DMI and saturation magnetization that allow the stabilization of skyrmionic ground states. An analysis of different contributions to the total magnetic free energy underlined critical issues influencing the skyrmions’ stability. Then, some manipulation strategies of the skyrmions’ core polarization and chirality by electric fields have been illustrated. Finally, we located the simulation window of parameters used in our extended modeling within the current experimental reality, in terms of existing or the perspective magnetic materials compatible with skyrmionic applications in classic, neuromorphic and quantum information technologies.

## Figures and Tables

**Table 1:** Selection of thin film multilayered materials illustrating Néel skyrmion stabilization. We indicate the material (multilayer system), the measured diameter of the skyrmion core, the magnitude of the DMI $\left|D\right|\left(\frac{mJ}{m^{2}}\right)$, the temperature of the skyrmion stability and the reference of the paper containing the study.

Multilayer System	Diameter of Skyrmion Core (nm)	$\left|D\right|\left(\frac{mJ}{m^{2}}\right)$	Temperature of Skyrmion Stability (K)	Reference
Pt/Co/Ta	75–200	1.3	≶300	[5]
Pt/Co/MgO	70–130	2.0	≶300	[6]
Ir/Co/Pt	25–100	N.A.	≶300	[7]
[Ir/Co/Pt]_10_	100	2	>300	[8]
Pt/CoFeB/MgO	<250	1.35	≶300	[9,10,11]
Pd/CoFeB/MgO	<200	0.78	≶300	[12]
W/CoFeB/MgO	250	0.3–0.7	≶300	[13]
Ta/CoFeB/MgO	300	0.33	≶300	[14]
Ta/CoFeB/Ta/MgO	1000–2000	0.33	>300	[14]

**Figure A1:**
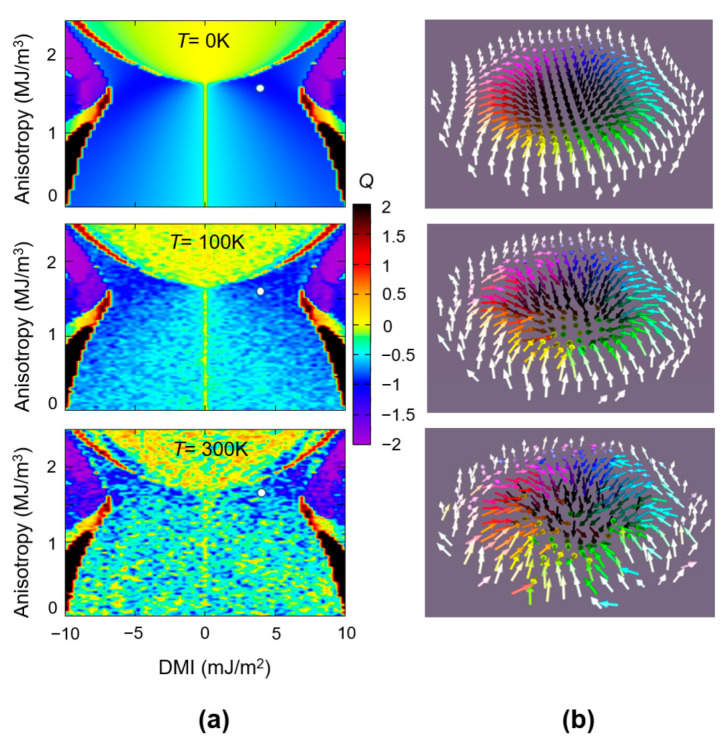
(**a**) *DMI*-*PMA*-*Q* phase diagrams calculated at different temperatures 0 K, 100 K, 300 K. (**b**) Magnetization vector field representation of skyrmionic structures corresponding to the point (*DMI* = 5 mJ/m^2^, *K_u_* = 1.7 × 10^+6^ J/m^3^) indicated with a white filled circle in the diagrams of the panel (**a**). The calculation has been performed for a 90 nm disk with the following fixed parameters: *M_s_* = 1714 kA/m, *A_ex_* = 2.1 × 10^+11^ J/m; the Gilbert damping constant *α* = 0.01.

**Figure A2:**
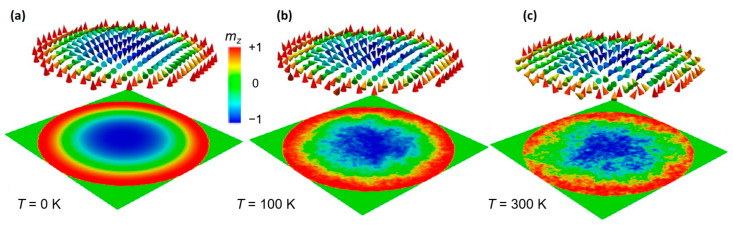
Skyrmionic state at different temperatures: (**a**) T = 0 K, (**b**) *T* = 100 K, (**c**) *T* = 300 K. The calculation has been performed for a 90 nm disk with the following parameters: *DMI* = 5 mJ/m^2^, *K_u_* = 1.7 × 10^+6^ J/m^3^, *M_s_* = 1714 kA/m, *A_ex_* = 2.1 × 10^+11^ J/m; the Gilbert damping constant *α* = 0.01. In each panel, the top image is a glyph representation of the magnetization vector field within the disk. The bottom image is a scalar field representation of the *m*_z_ values.

**Figure A3:**
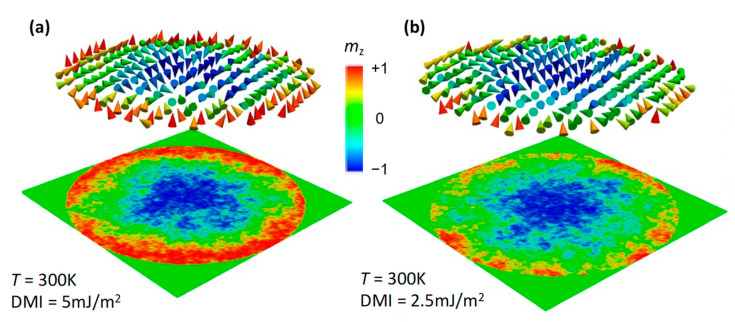
Skyrmionic state at *T* = 300 K for: (**a**) *DMI* = 5 mJ/m^2^ and (**b**) *DMI* = 2.5 mJ/m^2^. The calculation has been performed for a 90 nm disk with the following parameters: *DMI* = 5 mJ/m^2^, *K_u_* = 1.7 × 10^+6^ J/m^3^, *M_s_* = 1714 kA/m, *A_ex_* = 2.1 × 10^+11^ J/m; the Gilbert damping constant *α* = 0.01. In each panel, the top image is a glyph representation of the magnetization vector field within the disk. The bottom image is a scalar field representation of the *m*_z_ values.

## Data Availability

The data presented in this study are available on request from the corresponding author.

## Appendix A

### The Influence of Thermal Fluctuations at Finite Temperature

The ab-initio as well as the micromagnetic calculations presented so far have been performed at zero Kelvin. This simplified approach, which is not considering the thermal blur effects, allowed us to clearly observe and critically predict critical the magnetic properties and behavior. However, for the experimental extrapolation of the results, one may wonder about the thermal stability of the theoretically predicted chiral structures (e.g., skyrmions). To answer to this question, we have also performed sets of micromagnetic simulations at finite temperature. In the *Mumax3* code, the temperature is implemented by means of a fluctuating thermal field: ${\vec{B}}_{therm}=\vec{\eta }\left(step\right)\sqrt{\frac{2\mu_{0}\alpha k_{B}T_{sim}}{\gamma_{0}\mu_{0}M_{s}V_{sim}\Delta t}}$, where *α* -damping parameter, $k_{B}$ – Boltzmann constant, $T_{sim}$ -simulation temperature, $\gamma_{0}$ – gyromagnetic ratio, $\mu_{0}$ -magnetic permeability, $M_{s}$- saturation magnetization, $V_{sim}$ – the simulation volume (cell) and Δt- the simulation time step, $\vec{\eta }\left(step\right)$ is a random vector from a standard normal distribution whose value is changed every step.

As illustrated in Figure A1, with increasing the temperature, the thermal field is randomly disordering the local spins within a certain chiral structure. Figure A1, panel (a) shows the *DMI*-*PMA*-*Q* phase diagrams calculated at 0 K, 100 K and 300 K. The panel (b) illustrates a skyrmionic structure, corresponding to the white point in each diagram, for each temperature.

At a finite temperature, locally, the spin orientation will have a random distribution around the “equilibrium” zero-Kelvin position. The larger the temperature, the larger the width of this distribution. The temperature and DMI dependent random distribution of local spins orientation will further affect the value of the numerically calculated topological charge: $Q=\frac{1}{4\pi }\int d^{2}x\;\vec{m}\cdot \left(\frac{d\vec{m}}{dx}\times \frac{d\vec{m}}{dy}\right)$, as illustrated in Figure A1, panel (a).

A qualitative illustration concerning the stability in temperature of a skyrmionic structure, is also presented in Figure A2, in which vector field glyph representation is superimposed to corresponding scalar field *m*_z_ maps, for each temperature.

One can observe that, for the chosen value of the DMI and PMA, the skyrmionic structure survives even at room temperature (*T* = 300 K). However, for temperatures above 300 K, the skyrmionic state will finally be erased by the local thermal field. Because the barrier protecting the spins from the core to reverse their orientation is controlled by the DMI (as illustrated in Figure 12b), the thermal stability of the skyrmions is increasing with the DMI increase (as qualitatively illustrated in Figure A3), or by applying a magnetic field.
